# IgG based immunome analyses of breast cancer patients reveal underlying signaling pathways

**DOI:** 10.18632/oncotarget.26834

**Published:** 2019-05-28

**Authors:** István Gyurján, Sandra Rosskopf, Johana A. Luna Coronell, Daniela Muhr, Christian Singer, Andreas Weinhäusel

**Affiliations:** ^1^ Austrian Institute of Technology AIT, Center for Health & Environment, Molecular Diagnostics Unit, Vienna, Austria; ^2^ Department of Obstetrics and Gynecology, Medical University of Vienna, Vienna, Austria

**Keywords:** protein microarray, immunome, breast cancer, signaling pathways, differentially reactive antigens

## Abstract

**Background:** Breast cancer is the most frequent and one of the most fatal malignancies among women. Within the concept of personalized medicine, molecular characterization of tumors is usually performed by analyzing somatic mutations, RNA gene expression signatures or the proteome by mass-spectrometry. Alternatively, the immunological fingerprint of the patients can be analyzed by protein microarrays, which is able to provide another layer of molecular pathological information without invasive intervention.

**Results:** We have investigated the immune signature of breast cancer patients and compared them with healthy controls, using protein microarray-based IgG profiling. The identified differentially reactive antigens (n=517) were further evaluated by means of various pathway analysis tools. Our results indicate that the immune signature of breast cancer patients shows a clear distinction from healthy individuals characterized by differentially reactive antigens involved in known disease relevant signaling pathways, such as VEGF, AKT/PI3K/mTOR or c-KIT, which is in close agreement with the findings from RNA-based expression profiles.

**Conclusion:** Differential antigenic properties between breast cancer patients and healthy individual classes can be defined by serum-IgG profiling on protein microarrays. These immunome profiles provide an additional layer of molecular pathological information, which has the potential to refine and complete the systems biological map of neoplastic disease.

## INTRODUCTION

In recent decades great efforts have been made to reduce the incidences of breast cancer, which increases each year. External and internal risk factors, such as the ever-increasing environmental burden, inadequate lifestyles- or dietary habits, are thought to aggravate the current situation [1] and might eventually lead to somatic changes. With the advent of new diagnostic tools, such as ultrasound, magnetic resonance imaging and mammography screenings mortality rates have been slightly reduced [2]. Nevertheless, the low sensitivity or specificity of such techniques results in detection of breast cancer often at an advanced stage. Hence, there is an important need to develop new tools that would allow for early cancer detection and are less invasive (*e.g.* avoid unnecessary biopsies, irradiation by mammography). This is especially important when clinicians try to discriminate between malignant breast cancer and benign breast lesions. An ideal solution would be to identify cancer specific biomarkers from body fluids, such as blood, urine or saliva. Genomic and proteomic approaches including altered DNA methylation- or microRNA patterns or specific protein signatures could provide solutions for these requirements. Another option would be to detect tumor-associated antibodies specific to tumor antigens of patients, before (through screening regimes) or early at the onset of cancer development [3].

Cancer related antigens were first recognized in the 1950’s based on observations from chemically induced carcinomas in mice [4, 5, 6]. Ever since tumor-associated antigens have been reported in a variety of cancer entities, such as breast-, ovarian-, prostate- or colon cancer [7, 8, 9]. The immunogenicity of tumor-associated antigens has been attributed to aberrantly expressed proteins, which could derive from mutations, altered post-transcriptional or translational modifications, abnormal cellular localizations or deregulated apoptotic/necrotic processes [10, 11, 12]. From a diagnostic point of view, only one such an antigen is insufficient to discriminate between cases and controls because of lack of sensitivity and specificity, thus multiple antigens have to be used simultaneously. Distinction has to be made as well when classifying antigens whether those are common among most cancers or specific to only one type. As an example, damage-associated molecular patterns (DAMPs) are frequently associated with cancer, chronic inflammation and necrosis [13, 14, 15]. Therefore molecules, such as HSP90, HMGB1, S100 and mitochondrial DNA are frequently detected in diverse cancer-related diagnostic studies [16, 17, 18]. Finding a tumor specific autoantibody signature is even more complicated since there are large variations between patients in general. Breast cancer is also not an exception and it is largely heterogeneous in terms of structural-, molecular-, genomic-, intratumoral- and micro-environmental variations [19]. Inflammatory cells, endothelial cell, pericytes, tumor-associated fibroblasts, cancer cells and constituents of extracellular matrix can all display large diversity of antigens on their surface or upon disintegration. The situation becomes even more complex with cancer progression because of accumulating genetic and epigenetic alternations within cancer cells (*e.g.* increasing aneuploidy), yielding vast amount of aberrantly expressed proteins. Furthermore, along the course of tumor development/progression the temporal changes of immune competence (*i.e.* immune evasions) could alter the composition of tumor-associated antibodies.

In this study, we have investigated the circulating antibody (IgG) signature of non-hereditary breast cancer patients and compared them with healthy controls, using protein microarray analyses. The aim of this discovery-phase study was to identify breast cancer-associated antigens in order to use them as a potential tool for diagnostics in the future. We asked whether the circulating IgG antibody repertoire of breast cancer patients could reflect spectra of antigens and the biological phenomena they are involved in. Our results indicate that the immune signatures of breast cancer patients show a clear distinction from healthy individuals characterized by the biological pathways in which the corresponding antigens are participate.

## RESULTS

### Differentially reactive antigen data shows similarities with expression profiles of breast cancer

Seventy-seven IgG samples from non-hereditary breast cancer patients were screened on 16K protein microarray and compared with 62 IgG samples from healthy controls. The clinico-pathological data of study samples are shown in Table 1. The list of differentially reactive antigenic proteins consists of 516 entries, where 305 antigens were “upregulated” in cancer and 211 antigens reactivity were decreased (Supplementary Table 1).

**Table 1:** Clinico-pathological features of study samples

Characteristics	Cancer samples (n=77)	Control samples (n=62)
**Mean age** [years±SD]	54.8±15.3	76.9±7.7
**Tumor grade**		
G1; G2; G3	19;23;33	n/a
N/A	2	
**Estrogen receptor positive**	49	n/a
N/A	28	
**Progesterone receptor positive**	27	n/a
N/A	50	
**Her2/neu receptor positive**	26	n/a
**pN stage^d^**		n/a
pN0; pN1; pN1a; pN1b	38;5;7;2	
pN2; pN2a; pN3; pNX	1;5;4;9	
N/A	8	
**pT stage^d^**		n/a
pT1; pT1a; pT1b; pT1c; pT1mic	2;5;6;27;3	
pT2; pTis; pTx	16;12;1	
N/A	5	
**Metastasis stage^e^**		n/a
M0; M1; MX	19;7;6	
N/A	45	
**Menopause status^f^**		
Pre-menopause	23	
Post-menopause	47	62
N/A	7	
**Chemotherapy before sampling**	2	n/a

Abbreviations: n/a - not applicable or not available; ^d^pT stage and pN stage information from 71 patients: pT1a - Tumor less than 0.5 cm in greatest dimension; pT1b - Tumor more than 1.0 cm but not more than 1.0 cm in greatest dimension; pT1c - Tumor more than 1.0 cm but not more than 2.0 cm in greatest dimension; pT1mic - Microinvasion 0.1 cm or less in greatest dimension; pT2 - Tumor more than 2.0 cm but not more than 5.0 cm in greatest dimension; pTis - Carcinoma in situ; pN0 - No regional lymph node metastasis; pN1 - Metastasis to movable ipsilateral axillary lymph node(s); pN1a - Only micrometastasis (none larger than 0.2 cm); pN2 - Metastasis to ipsilateral axillary lymph node(s) fixed to each other or to other structures; pN2a - Metastasis in 4-9 axillary lymph nodes, including at least one that is larger than 2 mm; pN3 - Metastasis to ipsilateral internal mammary lymph node(s); pNX - Axillary lymph nodes cannot be assessed; M0 - No distant metastasis; M1 - Distant metastasis present (includes metastasis to ipsilateral supraclavicular lymph nodes); MX - Presence of distant metastasis cannot be assessed.

Having the analyzed IgG samples from sporadic breast cancer patients we scanned the COSMIC database to find concordance with protein entries associated with somatic alternations in breast cancer. Out of the 516 antigens 34 proteins were found (Supplementary Table 1) amongst the so-called COSMIC-census genes (n=572, at the time of the study), which considered the most important cancer genes (proteins) with somatic (or both somatic and germ-line) mutations. Meta-analysis also shows the percentage of breast cancer cases (in COSMIC database; n=883 samples) in which the given gene was up- or down regulated; or copy number variation was detected (n=761 samples) (Supplementary Table 1).

Furthermore, we have checked the overlap between various differentially expressed gene sets and our dataset using GSEA/MSig database, oncogenic- and immunologic signature option. Among the most significant overlaps we found up-regulated gene sets in MCF-7 breast cancer-, and MCF-10 mammary epithelium cell lines, where CCND1, MAP2K1 or EIF4G genes were over-expressed/knockdown, respectively (Table 2). Regarding the immunologic signature overlap the most significant gene set was that in which the effects of diabetes were measured on peripheral blood mononuclear cells. According to certain studies there is an association between diabetes and the risk of cancer development and outcome [20, 21].

**Table 2:** Overlap of genes from breast cancer *vs.* control comparison with gene sets from the Molecular Signature Database (MSigDB) within Gene Set Enrichment Analysis (GSEA) tool

A: Oncogenic signature overlaps			
Description	Genes in overlap/Genes in genset	p-value	FDR q-value
Genes up-regulated in MCF10 (mammary) cells vs. knockdown of EIF4G1 gene by RNAi.	10/95	1.03E-07	1.40E-05
Genes up-regulated in MCF-7 cells (breast cancer) over-expressing CCND1 gene.	13/188	1.97E-07	1.40E-05
Genes up-regulated in MCF-7 cells (breast cancer) over-expressing a mutant K112E form of CCND1 gene.	13/190	2.22E-07	1.40E-05
Genes up-regulated in SH-SY5Y cells (neuroblastoma) in response to PDGF stimulation.	11/146	7.31E-07	3.45E-05
Genes up-regulated in MCF-7 cells (breast cancer) positive for ESR1. MCF-7 cells stably over-expressing constitutively active MAP2K1 gene.	12/196	2.09E-06	7.90E-05
Genes up-regulated in granule cell neuron precursors (GCNPs) after stimulation with Shh for 24h.	11/183	6.60E-06	2.08E-04
Genes up-regulated in epithelial lung cancer cell line over-expressing an oncogenic form of KRAS gene.	11/193	1.09E-05	2.72E-04
Genes down-regulated in primary keratinocytes from RB1 skin specific knockout mice.	9/126	1.15E-05	2.72E-04
Genes down-regulated in HUVEC cells (endothelium) by treatment with VEGFA.	10/193	6.12E-05	1.21E-03
Genes up-regulated in NCI-60 panel of cell lines with mutated TP53	10/194	6.39E-05	1.21E-03
**B: Immunologic signature overlaps**			
Genes up-regulated in comparison of peripheral blood mononuclear cells from patients with type 1 diabetes at the time of diagnosis vs. those at 4 month later.	21/200	1.08E-14	1.03E-11
Genes up-regulated in peripheral blood mononuclear cells from patients with type 1 diabetes at the time of diagnosis vs. those with type 2 diabetes at the time of diagnosis.	21/200	1.08E-14	1.03E-11
Genes up-regulated in comparison of unstimulated CD8 T cells at 48 h vs. CD8 cells at 48 h after stimulation with IL12.	20/200	1.18E-13	7.54E-11
Genes up-regulated in comparison of control thymocytes vs. thymocytes treated with dexamethasone [PubChem=5743].	19/200	1.23E-12	5.85E-10
Genes down-regulated in comparison of unstimulated NK cells vs. those stimulated with IL2.	18/200	1.20E-11	1.50E-08
Genes down-regulated in comparison of IgD+ peripherial blood B cells vs. dark zone germinal center B cells.	17/200	1.10E-10	1.50E-08
Genes up-regulated in comparison of unstimulated peripheral blood mononuclear cells vs. those stimulated with YF17D vaccine.	17/200	1.10E-10	1.50E-08
Genes down-regulated in comparison of CD8 T cells at 0h vs. those at 48 h.	17/200	1.10E-10	1.50E-08
Genes up-regulated in comparison of NKT cells vs. monocyte macrophages.	17/200	1.10E-10	1.50E-08
Genes up-regulated in comparison of CD4 dendritic cells vs. CD4-, CD8- dendritic cells.	17/200	1.10E-10	1.50E-08

### Inferring signaling pathways from differentially reactive antigens

**Figure 1:**
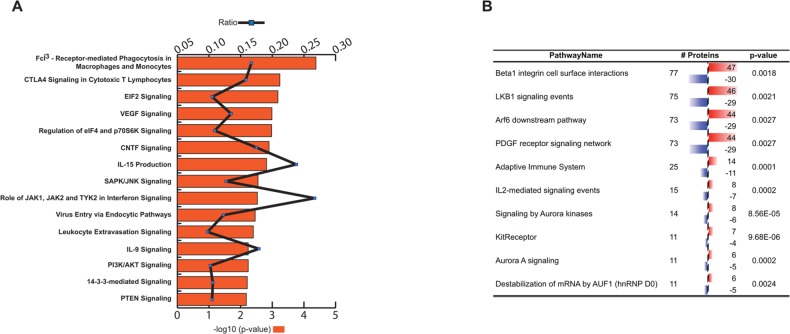
**(A)** Ingenuity global canonical pathways inferred from differentially reactive antigens. Minus-log10 p-values (bars) and enrichment ratios (line) are shown. **(B)** Deduced molecular pathways using PathwayCommons tool. Red bar indicates increased-, blue bar decreased antigen binding reactivity.

Ingenuity Global Canonical Pathways were assessed and the top 15 most significant pathways were plotted with the ratio of enrichment (Figure 1A). Supplementary Table 2A shows Ingenuity Canonical Pathway analyses results, threshold set at p<0.05. The top canonical pathways found are related to various immunological- and inflammatory processes, and to cancer. The most significant (p=4.17E-05) canonical pathway is the *Fcγ Receptor-mediated phagocytosis by macrophages and monocytes*. The major function of these receptors is to bind monomeric or aggregated IgG molecules, immune complexes or opsonized particles [22]. Upon receptor binding internalization of the complex is initiated with cup formation and subsequent phagosome development, which may finally lead to antigen presentation via activating or inhibitory pathways. These antigens could derive from the cellular constituents of dying macrophages, which are recruited to the sites of tumor, through various “danger signals” (*e.g.* HSP90 and HMGB2; upregulated in cancer in this dataset; Supplementary Table 1) [13] derived from the tumor itself. High numbers of resident or recruited macrophages at tumor sites are associated with elevated inflammation and poor outcome. Specifically, the found proteins are associated with actin cytoskeleton characteristic to phagocytosis. Tumor-associated macrophage functions are also related to *VEGF*- (p=1.02E-03), *PTEN*-(p=6.61E-03) and *mTOR*- (p=2.14E-02) signaling pathways, which are common examples for cancer related processes including tumor angiogenesis. In summary, most of the found Ingenuity canonical pathways are associated with the components of the PI3K/PTEN/Akt/mTOR pathway, which signaling cascade is estimated to be deregulated by gene mutations in more than 70% of all breast cancer [23]. Yet worth to mention, the canonical pathway *CTLA-4 Signaling in Cytotoxic T Lymphocytes* is also highly significant (p= 5.75E-04) through Ingenuity. Although the CTLA-4 (Cytotoxic T lymphocyte-associated antigen-4) protein itself is not among the differentially reactive antigens in our dataset the corresponding pathway serves as a good example on how an immune process counteracts with cancer [24]. Of note, another immune-checkpoint molecule LAG-3 (Lymphocyte activation gene-3) is found to be differentially antigenic and upregulated in the cancer group.

**Figure 2:**
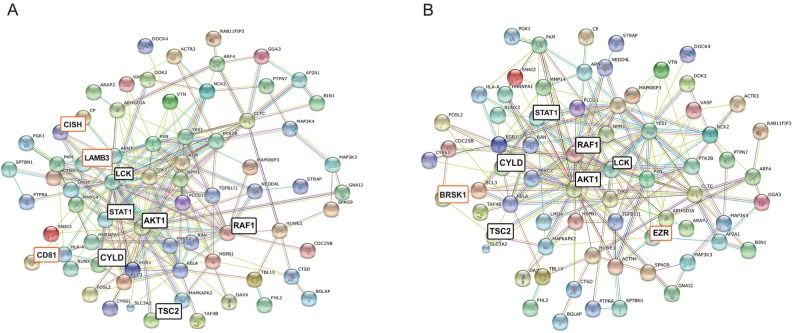
**(A)** Deduced molecular associations related to integrin-mediated interactions, based on PathwayCommons analysis. **(B)** Deduced molecular associations related to LKB1-mediated interactions. Disconnected nodes are not shown. Major core molecules are highlighted with black; differences between integrin- and LKB-1 related networks **(B)** are highlighted with orange.

Complementing the above results from other aspects we have analyzed our dataset by Pathway Commons as well, using Webgestalt integrated gene-set enrichment tool kit. Figure 1B shows the top 10 identified pathways in PathwayCommons analyses (Supplementary Table 2B). The highest numbers of antigens, 77 proteins, was associated with *Beta1 integrin cell surface interactions* (p= 0.0018). In the context of mammary gland development Beta1-integrins are essential for luminal polarity and myoepithelial contraction [25, 26]. Consequently, deregulated expression of integrins results in altered tissue architecture and metastasis of breast cancer [27, 28, 29]. In order to validate and visualize molecular associations (within this category) we have ran protein-protein interaction analysis (String Database). The 77 mapped proteins are predicted to interact with each other with high significance giving 178 observed interactions (p=6,11E-15) (Figure 2A). One of the central nodes is AKT1 with TSC2 and RAF1 axis; the other core is LCK (lymphocyte-specific protein-tyrosine kinase) with STAT signaling. LCK is a non-receptor protein-tyrosine kinase and has a fundamental role in T-cell receptor mediated signaling, thus thymocyte development and T-cell activation [30].

Yet another highly represented (75 proteins) pathway is the *LKB1 mediated signaling* (p=0,0021) (Figure 1B). The Liver Kinase B1 (LKB1) gene product is a serine/threonine kinase and has pleiotropic functions in cell growth, epithelial polarity and energy metabolism [31]. LKB1 is also considered as tumor suppressor that is lost in several cancer types [32] including breast cancer [33], and it is able to act through several signaling cascades, such as mTOR, AMPK (5' adenosine monophosphate-activated protein kinase) or PI3K/AKT [34]. Analyzing the molecular associations (String database), composition of the involved molecules in the core part of this network is very similar to the integrin signaling associated molecules mentioned above (only 6 molecules are different), resulting in a slightly higher number of interacting proteins (n=181, p=1.55E-15) (Figure 2B, Supplementary Table 2B). One unique protein within LKB1 pathway is Ezrin (EZR), which has been shown to mediate breast cancer cell migration, hence facilitating metastasis [35].

Regarding both Beta1-integrin mediated interactions and LKB1mediated signaling in breast cancer the CYLD (cylindromatosis) protein is of paramount importance too, which act as a deubiquitinating enzyme and is considered as a tumor suppressor [36, 37].

In the PathwayCommons analysis the signaling pathway with the highest significance (p= 9.68E-06) was the *Kit-receptor mediated signaling*. One of the interesting upregulated protein within this pathway is CISH (Cytokin-Inducible SH2-Containing Protein), which have been found as a key suppressor of IL15 and JAK signaling [38].

**Figure 3:**
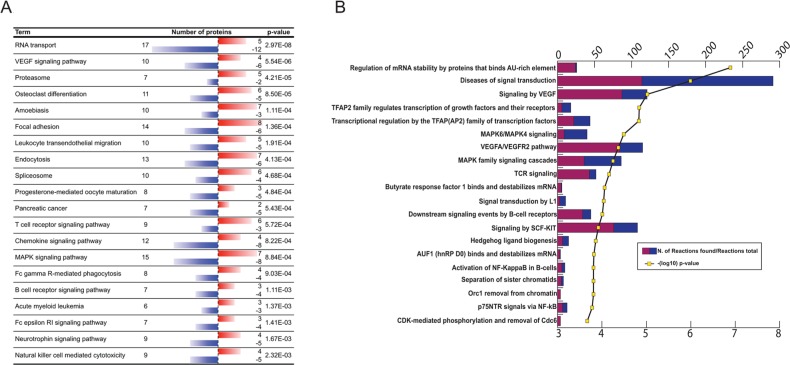
**(A)** KEGG pathway analysis of differentially antigenic proteins. Red bar indicates increased-, blue bar decreased antigen binding reactivity. **(B)** REACTOME pathway analysis of differentially antigenic proteins. Purple/blue bars represent the number of related reactions/all reactions in category; yellow line shows -(log10) p-values.

The KEGG pathway analyses (Figure 3A; p<0,0025) resulted in a similar profile of functions to that of Ingenuity, *i.e.* enrichment of proteins involved in cytoskeletal rearrangements during phagocytosis, VEGF- and proteasome pathways and immune cell functions. One of the exceptions from the similarities would be the *RNA transport* associated event. Molecules within these functions are involved in cytoplasmic transport of RNA, ribosomal binding of RNAs, pre-mRNA splicing, nucleopore complex formation and translation initiation and elongation. The KEGG analysis found 17 RNA transport associated molecules (Supplementary Table 2C) and 27 interactions were predicted by String with high significance (p=8.88e-16, Supplementary Figure 1).

The top 20 terms of Reactome analysis (p≤2.12E-04; FDR≤0,012; Supplementary Table 2D) reinforce some of the findings by Ingenuity, Pathway Commons or KEGG analysis: VEGF-, Kit-, TCR- and MAPK signaling (Figure 3B). The top ranked term “*Regulation of mRNA stability by proteins that binds AU-rich element*” (p-value=1,7E-7; FDR=2,05E-4) shows also consensus with the PathwayCommons result (“*Destabilization of mRNA by AUF1 (hnRNP D0)*”). In this process AU rich binding protein (AUF1) dimers bind to adenyl-uridyl-rich elements (ARE) elements of certain mRNAs’ UTR, recruiting additional proteins, such as Poly-A binding protein, heat-shock proteins, translation initiation factor eIF4G, which finally may lead to mRNA degradation [39].

Another important category regarding breast cancer is “*Transcriptional regulation by the AP-2 (TFAP2) family of transcription factors”*. The family members of AP-2 regulate the cell growth and differentiation of tissues of ectodermal origin and involved in the regulation c-erbB-2 (HER2) in breast cancer [40, 41].

**Figure 4:**
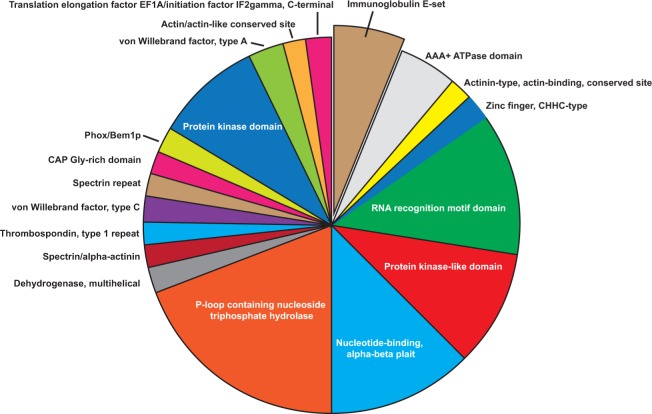
Enriched protein domains (n=84) of the differentially reactive proteins using InterPro database.

We also wanted to find enriched protein domains that are preferentially recognized by the IgG pool of breast cancer patients. Therefore, InterPro functional classification was performed using all proteins (Cancer vs. Ctrl) with changed immune-reactivity (Figure 4.). Ninety-eight proteins showed significantly enriched protein domains (p<0.05). We found that the protein domain with the highest relevance (p= 6.40E-04) was *Immunoglobulin E-set domain*, and the most abundant domains were *P-loop containing nucleoside triphosphate hydrolase* (n=19; p=2.19E-2) and *RNA recognition motif domain* (n=12; p=1.45E-2). Importantly, *Protein kinase-like domains*, which also include the protein kinase domain subgroup, are also enriched (p= 1.82E-02) in our dataset. Proteins with these domains are highly conserved and have fundamental role in cell proliferation, apoptosis and differentiation [42].

**Figure 5:**
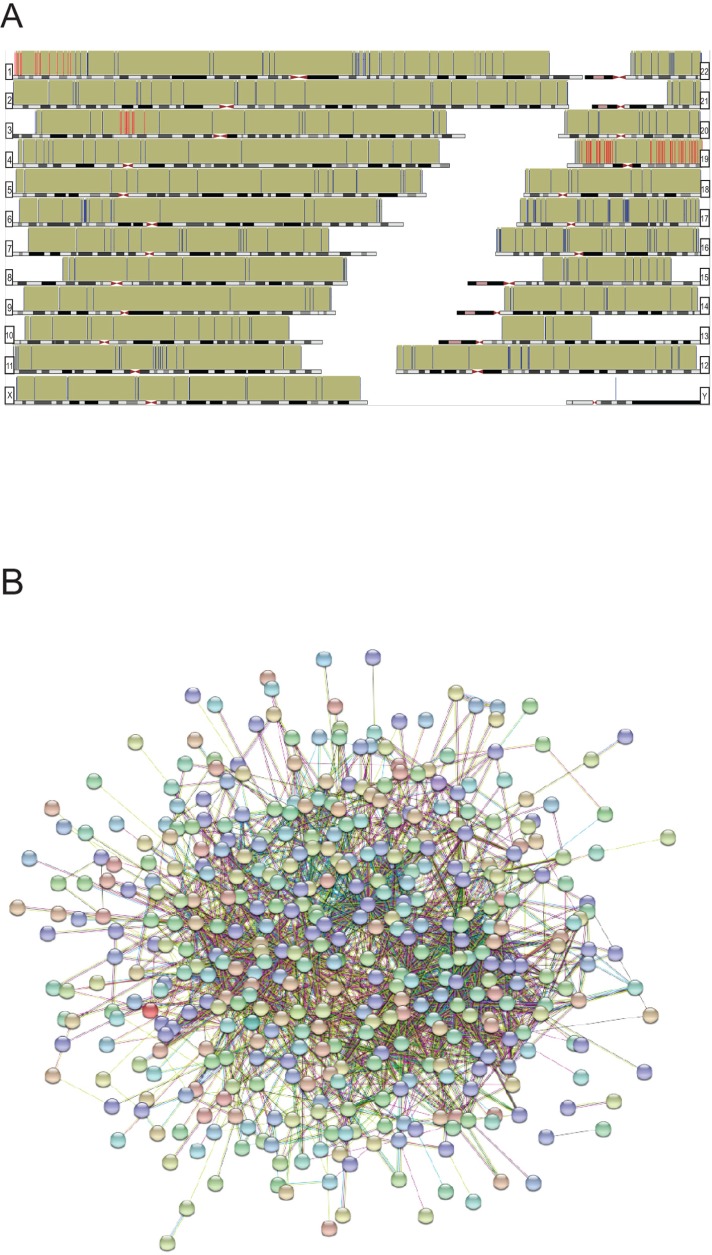
**(A)** Genomic distribution of the differentially reactive antigens (hg38). Chromosomal locations are represented with bars; red bars indicate enriched regions, according to GSEA database. **(B)** Deduced protein-protein associations of all mapped (n=502) differentially reactive proteins, based on String database. Only connected nodes are shown as bubbles.

Finally, we analyzed whether the protein data set was enriched at certain chromosomal locations, *i.e.* to find possible foci that could be mutational hotspots/targeted by overexpressed or mutated proteins. According to GSEA (Gene Set Enrichment Analysis) we have found 4 enriched genomic regions: Chr3p21 (14 genes, p=2.36E-06); Chr19q13 (27 genes, p=9.21E-06); Chr19p13 (20 genes, p=4.15E-05) and Chr1p36 (15 genes, p=5.63E-04). (Figure 5A; Supplementary Table 3).

In summary, 502 of 516 differentially reactive protein entries were mapped using String database. These proteins are predicted to be involved in 1968 interactions with each other (p=9.77E-14) (Figure 5B.). It also suggests that the differentially reactive antigenic protein based immune profiles of breast cancer patients are able to elucidate protein associations/complexes too, and could directly reflect the underlying signaling pathways accompanied with breast cancer development.

## DISCUSSION

In this discovery-study, we have used high-throughput protein microarray to identify altered immune-reactivity based on breast cancer patients’ IgG profile. Recognizing immune-phenotypes by different patient groups not only helps to develop tools for early-stage breast cancer detection, but also allow to get insight into molecular pathways and to devise potential new targets.

Breast cancer, as other tumor types, is not only heterogeneous by means of patients but also consists of different cell types in the tumor microenvironment. Inflammatory cells, endothelial cell, pericytes, tumor-associated fibroblasts, cancer cells and constituents of extracellular matrix can all display large diversity of antigens on their cell-surface or upon disintegration. The situation becomes even more complex with cancer progression because of accumulating genetic and epigenetic alterations within cancer cells (e.g. increasing aneuploidy), yielding vast amount of aberrantly expressed proteins. Providing that the immune system can recognize these tumor-associated antigens as “non-self”, elimination of cancer cells may occur by cytotoxic T-lymphocytes and natural killer cells, as the best scenario. Unfortunately, through the so-called immune editing mechanism [43], cancer cells are capable of escaping from detection and destruction. Looking at the comparison of IgG profile between the cancer and control group, we could see signs of both escape and elimination events. Related to this the CTLA-4 mediated signaling in CTLs refers to the negative regulation of T-cell function [24], which is clearly unfavorable in the case of cancer. Known as the earliest identified immune-checkpoint molecule, blocking CTLA-4 binding to its ligand with monoclonal antibodies has been recently approved by FDA in melanoma therapy [44]. Similarly to CTLA-4, LAG-3 also belongs to the immunoglobulin superfamily and negatively regulates T cell function. Since the administration of anti-LAG-3 antibodies were shown to inhibit Treg mediated immune suppression [45, 46] LAG-3 has become an attractive new target for immune-therapy.

Analyses revealed differentially reactive proteins involved in tumor angiogenesis (i.e. VEGF) as well, which is considered as an important hallmark of cancer [35]. Both tumor cells and immune-inflammatory cells (*e.g.* macrophages) are capable to emit vascular endothelial growth factor that can induce tumor-promoting angiogenesis. Moreover, matrix-bound latent VEGF ligand can be released by proteases responsible for degrading extracellular matrix [47]. Furthermore, mTOR and PTEN pathways are identified. Those are strongly associated with the AKT/PI3P signal transduction circuitry and frequently overexpressed (mTOR) or inactivated (PTEN) in a variety of cancer, including breast cancer [48, 49].

The presence of immune inflammatory cells at tumor sites is now a hallmark of cancer development [35]. The enhanced macrophage activity may refer to an increased clearance of apoptotic/necrotic cells or cell-debris but could also mean increased number of tumor-associated macrophages (TAM). These macrophages facilitate angiogenesis and matrix remodeling, and eventually promote tumor growth and metastasis as they have been found to be associated with poor prognosis in several cancer entities [50]. Increased levels of interleukin signaling (p= 7.41E-03) and leukocyte extravasation (p= 3.98E-03) from our dataset (Supplementary Table 2A) are also an indication of inflammatory situations; however, it is difficult to make conclusions how the tumor antagonizing or tumor promoting events take place.

One of the highly represented (75 entries) pathway involves LKB1 mediated signaling (p=0,0021, Figure 1B). Concerning the function in epithelial polarity LKB1 is involved in the mechanism of contact inhibition since suppression of its expression destabilize epithelial integrity and the cell became susceptible to Myc-induced transformation [35, 51, 52]. Analysis of the molecular association composition identified Ezrin (EZR) as a unique protein within LKB1 pathway (Figure 2B). The actin microfilament-associated EZR is a key regulator of Src activity [53, 54, 55] and has important function in tumor induced angio-/lymph angiogenesis [56].

Also, CYLD protein was identified both in Beta1-integrin mediated interactions and LKB1 mediated signaling in breast cancer samples. Main function of CYLD is to negatively regulate TNFR-mediated activation of NF-kappa-B signaling, and thus modulates inflammation. Additionally, it can affect cell division/proliferation through signaling pathways, such as Akt, MAPK or Wnt/Beta-Catenin [36]. Loss of CYLD up-regulates NFKB signaling and enhance metastasis in breast cancer [37].

Pathway Commons analysis identified the Kit-receptor mediated signaling pathways with the highest significance (p=9.68E-06). The c-Kit receptor is also a member of receptor tyrosine-kinase family, which upon binding with its ligand stem cell factor (SCF) could regulate apoptosis, proliferation, differentiation, and cell-motility in a variety of blood cells (red blood cell, T-cells, mast-cells). It has also important function in melanin- and gamete- formation, and Cajal-cells function of the gastrointestinal tract [57, 58]. Moreover, c-Kit over-expression was frequently found in triple negative breast cancer although its precise role in breast cancer development is still uncovered [59].

Yet another signaling cascade that is not discussed so far but also identified by the PathwayCommons test is the Aurora-A pathway (Figure 1B). It has been shown that Aurora-A is necessary for mitotic entry and centrosome separation, and is frequently overexpressed in various cancer types, including breast cancer, but not in benign breast lesions [60]. Mice carrying an MMTV-Aurora-A transgene activate the AKT/mTOR signaling pathways (with high level of CCND1) and are characterized by centrosome amplification, chromosome tetraploidization and premature sister chromatid segregation in the affected cells [61]. In addition to its oncogenic activity, Aurora A has been described as a tumor suppressor as well, which in turn has complicated recent efforts to develop inhibitors against this pleiotropic protein [62].

Taking together the results derived from the comparison of the IgG profile of breast carcinomas and control samples, it was found that those mostly related to the AKT/PI3K/mTOR pathway together with PTEN, complemented with c-Kit and Aurora A-signaling. These are then driving deregulated/changed integrin-, LKB1-and VEGF signaling that might lead to impaired cell-adhesion, contact inhibition and vascularization. The high prevalence of infiltrating immune cells might be indicative of an inflammatory situation during which crosstalk between leukocytes and cancer cells shaping the immune response. Also, the high degree of predicted protein-protein interactions shows the complexity of the underlying molecular pathways and the interconnection of signaling circuits.

## MATERIALS AND METHODS

### Clinical/Study samples

Samples were stored at -80°C until further utilization on protein microarray.

A total of 139 blood samples were collected, 77 of which were collected from patients with breast cancer: invasive ductal carcinoma (n=53), invasive lobular carcinoma (n=7), non-invasive intraductal carcinoma (n=14) and unknown type (n=3). Sixty-two blood samples were collected from healthy volunteers with no individual or familial history of breast or ovarian cancer. Detailed clinical data can be found as Supplementary Table 4.

### 16k protein microarray generation and processing

The in-house printed 16k protein microarray comprised of 6369 distinct proteins from which 5449 have been annotated with a gene-symbol. The recombinant proteins (each represented by 2 to 3 clones) derived from the UniPex expression libraries (human fetal brain, T-cell, lung- and colon expression libraries), developed at the former RZPD (Deutsche Ressourcenzentrum für Genomforschung), Berlin. Detailed description of the 16K protein microarray generation can be found in earlier report [63, 64, 65].

The protein array platform, the technical procedures for sample- and array processing and the application and advantages of purified IgG over raw serum or plasma samples were described previously [63, 66, 67]. Briefly, IgG purification of all serum samples was performed according to the manufacturer’s instructions using the Melon^TM^ Gel IgG Purification Spin Plate Kit (Thermo Scientific, Waltham, MA, USA). Quantifications of IgG samples were performed using the Epoch Microplate Spectrophotometer (BioTek Instruments, Winooski, VT, USA) to ensure their application on the microarrays at the same concentration (0.3 mg/mL). Integrity of the IgG samples were checked using SDS-PAGE on pre-casted NuPAGE^®^ Novex 4-12% Bis-Tris gels (Life Technologies, Carlsbad, CA, USA).

Protein microarray slides were blocked with DIG Easy Hyb solution (Roche, Basel, Swiss). Purified IgG was diluted to 0.3 mg/mL in two steps: first Melon^TM^ Gel Purification Buffer (Thermo Scientific) to normalized concentration of 0.6 mg/mL IgG and then 1:1 with 2x PBS with 0.2% Triton X-100 and 6% milk powder. The arrays were incubated for 4 h with constant rotation (12 rpm) at room temperature in a microarray hybridization oven (Agilent). After hybridization the slides were washed three times with PBSTx for 5 min, then incubated for 1 h with Alexa Fluor^®^ 647 goat anti-human IgG detection antibody (Invitrogen, Life Technologies). Slides were scanned using 10 µm resolution and 70% PMT to acquire array images.

### Data acquisition and statistical analyses

The scanned array images were imported into the GenePix Pro Microarray Acquisition & Analysis Software 6.0 (Molecular Devices, Sunnyvale, CA, USA) and the resulting fluorescence intensities of all features were calculated. The local background was subtracted from the median values before statistical data analysis, which was performed using R 2.10.0 and BRB-Array Tools 4.2.1 (https://linus.nci.nih.gov/BRB-ArrayTools.html) [68]. The log2 transformed intensity data were quantile normalized and filtered to exclude those features where less than 20% of expression data have at least 1.75-fold change in either direction from the gene’s median value (BRB). Additionally, data were filtered for minimum intensity of 256 (log_2_=8).

Differentially reactive antigens were determined using class comparison analyses (BRB-Array Tools) [68] at the significance thresholds for univariate tests of p≤0.001 and minimum fold changes of 1.5 between groups. Each batch was analyzed separately since our previous experiments [64] showed that high variances could exist between batches, which introduce non-biological differences; furthermore, batch-wise normalizations were also omitted since those could distort expression data. Summary table of differentially reactive antigens, Uniprot accession numbers, fold changes, p-values and False Discovery Rates (FDR) are shown in Supplementary Table 1.

Quantitative trait analysis was executed in BRB Array Tools to test whether patients age was correlated with the expression of differentially reactive antigens. Summary table can be found as Supplementary Table 5.

Differentially reactive protein names were submitted and analyzed with Ingenuity (https://www.qiagen.com/ingenuity) using Global Canonical Pathways (GCP) tool, which utilize right-tailed Fisher’s exact test for p-value calculations. Pathway-commons analyses were implemented in Webgestalt (Web-based Gene-set Analysis Toolkit; http://webgestalt.org) [69, 70, 71], which incorporate various tools different from Ingenuity. Webgestalt uses hypergeometric test for enrichment analyses and for each analysis the top 10 most significant categories were selected (as at least 2 proteins per category). Comparing identified differentially reactive antigens (as gene set) with other publicly available microarray results we used Molecular Signature Database (MSigDB) within GSEA, where oncogenic and immunologic signatures were tested (http://software.broadinstitute.org/gsea/index.jsp) [72, 73]. Cytogenetic mapping (enrichment) analysis was also performed in GSEA, then UCSC table browser and Genome graph tools were used to illustrate genomic locations.

For KEGG (Kyoto Encyclopedia of Genes and Genomes), Reactome, Interpro protein domain-based functional classification and protein-protein interactions analysis and visualization we have utilized String (Search Tool for the Retrieval of Interacting Genes/Proteins; https://string-db.org/) protein interaction database or through Reactome website (https://reactome.org) [74, 75, 76, 77].

In each analysis, as background proteins (*i.e.* random entries), we have used all annotated proteins presented on the 16k protein microarray; except for cytogenetic map analyses (GSEA) where the whole human genome (as gene-positions) was applied (Genome Reference Consortium Human Build 38).

## CONCLUSIONS

Detecting tumor-associated antibodies represents a highly attractive way for early diagnostics of cancer entities. Earlier discovery studies explain the underlying biological phenomenon as the immune response against mutated or aberrantly expressed proteins, which results in elevated reactivity. We think that, on one hand, the majority of identified antigens derive from the clearance of necrotic cells, which upon dying release large number of “targets” for the immune system, regardless if mutated or not. These events are complemented with the degradation of extracellular matrix, which not only emit ECM (Extracellular Matrix) constituents into the tumor microenvironment/circulation, but triggers sequestered growth factors. On the other hand, the observed reduced reactivity can be explained by the immune suppressive environment, orchestrated by the tumor cells. Despite the above potential explanations, due to high complexities of molecular interactions, it is difficult to figure out why reactivity of certain proteins was increased or decreased in cancer samples. Nevertheless, findings from our study reflect that the underlying signaling pathways are highly comparable to and complementing the information derived from gene expression profiling experiments of tumor tissue samples. In conclusion, inferring molecular pathways from antibody profiling provides a new molecular pathological layer of information associated with the health status of patients. Besides, the identified differentially antigens can be further analyzed as potential biomarkers in diagnostic tests or as immune therapy targets.

## SUPPLEMENTARY MATERIALS FIGURE AND TABLES










