# Supplementary material for: IgG based immunome analyses of breast cancer patients reveal underlying signaling pathways
Source: Oncotarget. 2019 May 28;10(37):3491–505. doi: 10.18632/oncotarget.26834 (PMC6544406; doi:10.18632/oncotarget.26834)
Supplement: Supplementary file 1 [file oncotarget-10-3491-s001.pdf]

# IgG based immunome analyses of breast cancer patients reveal underlying signaling pathways

## SUPPLEMENTARY MATERIALS

**Supplementary Table 1: List of differentially antigenic proteins**

**See Supplementary File 1**

**Supplementary Table 2: Complete list of genes associated with signaling pathways and terms mentioned in the article**

**See Supplementary File 2**

**Supplementary Table 3: Genes of the enriched genomic regions, according to GSEA analysis**

**See Supplementary File 3**

**Supplementary Table 4: Clinical features of breast cancer patients**

**See Supplementary File 4**

**Supplementary Table 5: Number of proteins whose expression was found to be correlated with age, using Spearmann correlation ($p \leq 0.001$), by testing differentially reactive antigens (n=516)**

| | Run1 | Run2 | Run3 | Run4 | Run5 | Run6 |
|---|---|---|---|---|---|---|
| Cases | 0 | 0 | 0 | 2 | 0 | 0 |
| Ctrl | 2 | 0 | 0 | 22 | 0 | 0 |

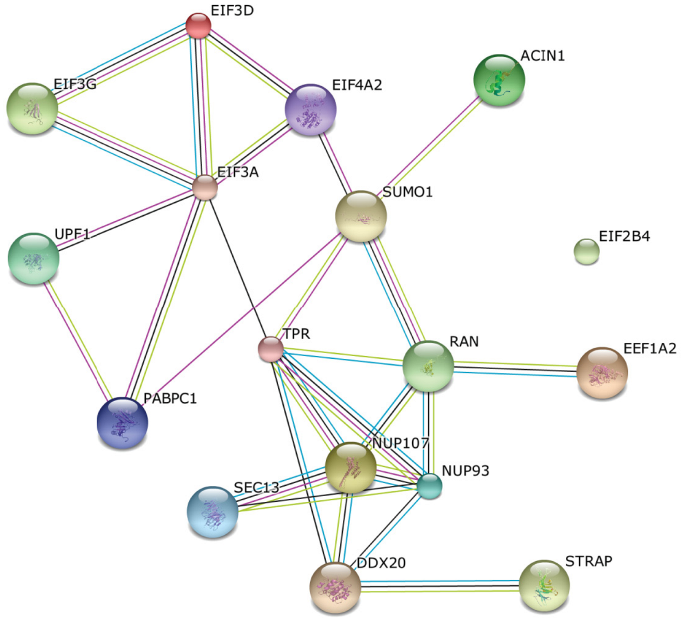


**Supplementary Figure 1: Deduced protein-protein interactions of the KEGG category "RNA transport" using String database (n=17; p=8.88e-16).**
