# Supplementary material for: IgG based immunome analyses of breast cancer patients reveal underlying signaling pathways
Source: Oncotarget. 2019 May 28;10(37):3491–505. doi: 10.18632/oncotarget.26834 (PMC6544406; doi:10.18632/oncotarget.26834)
Supplement: Supplementary file 2 [file oncotarget-10-3491-s002.docx]

**Supplementary Table 1: List of differentially antigenic proteins**

|  |  |  |  |  |  |  | **COSMIC DATA** | | | |  |  |
| --- | --- | --- | --- | --- | --- | --- | --- | --- | --- | --- | --- | --- |
| **Symbol** | **UniProt AccNo.** | **Fold changes** | **Up in Cancer** | **Parametric**  **p-value** | **FDR** | **Gene Census** | **Copynumber variations** | | | **GeneExpression** | | |
|  |  |  |  |  |  |  | **TotalN_**  **tested** | **CNV Gain %** | **CNV_Loss%** | **Tested** | **Overexpressed%** | **Underexpressed%** |
| **ADAMTS10** | **Q9H324** | **6.33** | **🡹** | **7.00E-07** | **0.000223** |  | **145** | **1.38** | **1.38** | **883** | **2.27** | **N/A** |
| **ATIC** | **P31939** | **5.3** | **🡹** | **5.10E-06** | **0.000727** | **+** | **761** | **0.26** | **N/A** | **883** | **5.89** | **0.57** |
| **ADAMTS16** | **Q8TE57** | **5.17** | **🡹** | **4.00E-07** | **7.79E-05** |  | **761** | **1.18** | **0.26** | **883** | **4.46** | **N/A** |
| **LCK** | **P06239** | **5.27** | **🡹** | **7.00E-07** | **0.000223** | **+** | **761** | **0.39** | **0.26** | **883** | **2.49** | **N/A** |
| **ANXA6** | **P08133** | **5.03** | **🡹** | **0.0006177** | **0.0432** |  | **761** | **0.13** | **0.53** | **883** | **2.38** | **1.02** |
| **CORO2B** | **Q9UQ03** | **4.92** | **🡹** | **1.00E-06** | **0.000276** |  | **761** | **0.53** | **0.39** | **883** | **1.93** | **N/A** |
| **SPTBN1** | **Q01082** | **4.69** | **🡹** | **< 1e-07** | **< 1e-07** |  | **761** | **0.66** | **N/A** | **883** | **5.1** | **N/A** |
| **AFMID** | **Q63HM1** | **4.75** | **🡹** | **6.00E-07** | **0.000223** |  | **761** | **3.15** | **N/A** | **883** | **2.6** | **N/A** |
| **PLEKHO1** | **Q53GL0** | **4.45** | **🡹** | **0.0003356** | **0.00862** |  | **761** | **5.78** | **N/A** | **883** | **2.6** | **N/A** |
| **CALCOCO2** | **Q13137** | **4.28** | **🡹** | **2.97E-05** | **0.00254** |  | **761** | **5.39** | **0.13** | **883** | **15.18** | **N/A** |
| **MCM7** | **P33993** | **4.28** | **🡹** | **3.80E-06** | **0.000654** |  | **761** | **0.26** | **0.39** | **883** | **6.34** | **N/A** |
| **RPL24** | **P83731** | **4.36** | **🡹** | **0.0001594** | **0.00544** |  | **761** | **0.66** | **N/A** | **883** | **3.51** | **N/A** |
| **CD81** | **P60033** | **4.3** | **🡹** | **4.00E-07** | **7.79E-05** |  | **761** | **N/A** | **0.53** | **883** | **3.06** | **N/A** |
| **RAB11FIP3** | **O75154** | **4.17** | **🡹** | **1.00E-07** | **3.51E-05** |  | **761** | **4.86** | **0.13** | **883** | **13.59** | **1.02** |
| **TUBA1B** | **P68363** | **4.16** | **🡹** | **1.91E-05** | **0.00203** |  | **761** | **0.26** | **0.13** | **883** | **8.04** | **N/A** |
| **MMP14** | **P50281** | **4.25** | **🡹** | **6.00E-07** | **0.000223** |  | **761** | **0.79** | **0.13** | **883** | **2.72** | **N/A** |
| **KIF22** | **Q14807** | **4.05** | **🡹** | **5.00E-06** | **0.000835** |  | **761** | **2.63** | **N/A** | **883** | **13.36** | **2.63** |
| **HSP90AB1** | **P08238** | **3.95** | **🡹** | **< 1e-07** | **< 1e-07** | **+** | **761** | **1.84** | **0.13** | **883** | **10.53** | **N/A** |
| **RCL1** | **Q9Y2P8** | **3.99** | **🡹** | **0.0004121** | **0.00979** |  | **761** | **1.05** | **0.92** | **883** | **7.93** | **1.02** |
| **FAM32A** | **Q9Y421** | **4.04** | **🡹** | **1.63E-05** | **0.00179** |  | **761** | **0.66** | **N/A** | **883** | **6.68** | **0.23** |
| **ENO3** | **P13929** | **4.01** | **🡹** | **2.71E-05** | **0.00254** |  | **761** | **1** | **4** | **883** | **0.45** | **N/A** |
| **ZNF7** | **P17097** | **3.78** | **🡹** | **4.80E-06** | **0.000709** |  | **761** | **10.25** | **N/A** | **883** | **35.11** | **2.38** |
| **HNRNPUL1** | **Q9BUJ2** | **3.78** | **🡹** | **< 1e-07** | **< 1e-07** |  | **761** | **0.53** | **0.13** | **883** | **8.81** | **1.13** |
| **CUL9** | **Q8IWT3** | **3.84** | **🡹** | **1.10E-05** | **0.0012** |  | **761** | **1.58** | **0.13** | **883** | **4.64** | **4.08** |
| **LMF2** | **Q9BU23** | **3.91** | **🡹** | **2.32E-05** | **0.00184** |  | **761** | **0.39** | **0.66** | **883** | **2.49** | **N/A** |
| **GABRA2** | **P47869** | **3.9** | **🡹** | **7.20E-06** | **0.000887** |  | **761** | **0.26** | **0.39** | **883** | **2.15** | **N/A** |
| **WDTC1** | **Q8N5D0** | **3.89** | **🡹** | **4.50E-06** | **0.000689** |  | **761** | **N/A** | **0.39** | **883** | **2.15** | **9.17** |
| **LEPREL1** | **Q8IVL5** | **3.88** | **🡹** | **9.39E-05** | **0.00597** |  | **761** | **1.71** | **N/A** | **883** | **0.79** | **N/A** |
| **TSC2** | **P49815** | **3.65** | **🡹** | **4.00E-07** | **0.000163** | **+** | **761** | **5.26** | **N/A** | **883** | **9.97** | **N/A** |
| **STOML2** | **Q9UJZ1** | **3.76** | **🡹** | **5.43E-05** | **0.00284** |  | **761** | **0.66** | **N/A** | **883** | **9.63** | **N/A** |
| **E4F1** | **Q66K89** | **3.76** | **🡹** | **< 1e-07** | **< 1e-07** |  | **761** | **5.12** | **N/A** | **883** | **6.34** | **N/A** |
| **DOK2** | **O60496** | **3.68** | **🡹** | **1.02E-05** | **0.00119** |  | **761** | **N/A** | **4.99** | **883** | **1.93** | **N/A** |
| **HARS** | **P12081** | **3.65** | **🡹** | **3.50E-06** | **0.000646** |  | **761** | **0.13** | **0.39** | **883** | **1.7** | **5.1** |
| **GDAP1L1** | **Q96MZ0** | **3.77** | **🡹** | **5.00E-07** | **0.000163** |  | **761** | **2.1** | **N/A** | **883** | **1.13** | **N/A** |
| **SAYSD1** | **Q9NPB0** | **3.53** | **🡹** | **2.48E-05** | **0.00193** |  | **761** | **1.19** | **0.13** | **N/A** | **N/A** | **N/A** |
| **RPL7** | **P18124** | **3.53** | **🡹** | **3.88E-05** | **0.00236** |  | **761** | **14.45** | **N/A** | **883** | **13.59** | **N/A** |
| **ACP1** | **P24666** | **3.51** | **🡹** | **4.99E-05** | **0.00279** |  | **761** | **0.26** | **0.26** | **883** | **8.38** | **0.79** |
| **ASF1B** | **Q9NVP2** | **3.57** | **🡹** | **0.0001117** | **0.00448** |  | **761** | **0.66** | **N/A** | **883** | **6.57** | **N/A** |
| **SLC7A5** | **Q01650** | **3.53** | **🡹** | **1.00E-07** | **5.30E-05** |  | **761** | **0.26** | **1.05** | **883** | **5.21** | **N/A** |
| **ZNF431** | **Q8TF32** | **3.55** | **🡹** | **7.30E-05** | **0.00343** |  | **761** | **0.66** | **N/A** | **883** | **4.98** | **0.11** |
| **SHKBP1** | **Q8TBC3** | **3.54** | **🡹** | **8.45E-05** | **0.00576** |  | **761** | **1.31** | **N/A** | **883** | **4.76** | **N/A** |
| **FOXL1** | **Q12952** | **3.53** | **🡹** | **1.00E-07** | **5.30E-05** |  | **761** | **0.26** | **1.45** | **883** | **4.08** | **N/A** |
| **PSMA2** | **P25787** | **3.45** | **🡹** | **0.0003566** | **0.0129** |  | **761** | **1.05** | **N/A** | **883** | **11.55** | **N/A** |
| **LAG3** | **P18627** | **3.47** | **🡹** | **0.0001378** | **0.00776** |  | **761** | **2.76** | **N/A** | **883** | **6.91** | **N/A** |
| **GTF2IRD1** | **Q9UHL9** | **3.39** | **🡹** | **1.11E-05** | **0.00353** |  | **761** | **0.79** | **0.13** | **883** | **6.46** | **0.11** |
| **VASP** | **P50552** | **3.46** | **🡹** | **4.10E-06** | **0.000668** |  | **761** | **0.66** | **0.13** | **883** | **6.46** | **0.11** |
| **TTC21B** | **Q7Z4L5** | **3.5** | **🡹** | **0.0001172** | **0.00453** |  | **761** | **0.13** | **N/A** | **883** | **5.32** | **N/A** |
| **FBXO34** | **Q9NWN3** | **3.46** | **🡹** | **3.05E-05** | **0.0037** |  | **761** | **0.53** | **0.13** | **883** | **4.53** | **2.49** |
| **BCL3** | **P20749** | **3.4** | **🡹** | **0.0009741** | **0.0251** | **+** | **761** | **0.92** | **0.13** | **883** | **3.74** | **N/A** |
| **CTDP1** | **Q9Y5B0** | **3.45** | **🡹** | **9.45E-05** | **0.00597** |  | **761** | **0.39** | **0.92** | **883** | **3.62** | **0.68** |
| **PBRM1** | **Q86U86** | **3.5** | **🡹** | **0.0001169** | **0.00453** | **+** | **761** | **0.13** | **0.53** | **883** | **2.72** | **1.02** |
| **RCBTB1** | **Q8NDN9** | **3.49** | **🡹** | **0.0001371** | **0.00776** |  | **761** | **N/A** | **2.23** | **883** | **2.49** | **1.36** |
| **SPTAN1** | **Q13813** | **3.42** | **🡹** | **6.30E-06** | **0.00084** |  | **761** | **0.13** | **N/A** | **883** | **2.27** | **0.68** |
| **EHD4** | **Q9H223** | **3.5** | **🡹** | **0.0002967** | **0.0114** |  | **761** | **0.39** | **0.26** | **883** | **1.7** | **1.93** |
| **IRF2BP2** | **Q7Z5L9** | **3.35** | **🡹** | **0.0006841** | **0.0142** |  | **761** | **9.86** | **N/A** | **883** | **18.23** | **0.23** |
| **WRNIP1** | **Q96S55** | **3.29** | **🡹** | **6.61E-05** | **0.00329** |  | **761** | **0.39** | **0.53** | **883** | **11.55** | **0.23** |
| **LMO4** | **P61968** | **3.37** | **🡹** | **0.0002087** | **0.00644** |  | **761** | **0.26** | **N/A** | **883** | **7.81** | **N/A** |
| **KRR1** | **Q13601** | **3.32** | **🡹** | **0.0001268** | **0.00485** |  | **761** | **1.31** | **N/A** | **883** | **7.47** | **0.34** |
| **NCK2** | **O43639** | **3.28** | **🡹** | **0.000612** | **0.0192** |  | **761** | **0.39** | **N/A** | **883** | **6.91** | **N/A** |
| **PRPF31** | **Q8WWY3** | **3.28** | **🡹** | **2.63E-05** | **0.00608** |  | **761** | **1.84** | **0.13** | **883** | **5.44** | **N/A** |
| **EEFSEC** | **P57772** | **3.32** | **🡹** | **3.60E-06** | **0.000848** |  | **761** | **0.53** | **0.13** | **883** | **4.87** | **0.45** |
| **AEBP1** | **Q8IUX7** | **3.35** | **🡹** | **1.00E-07** | **5.30E-05** |  | **761** | **1.18** | **N/A** | **883** | **4.08** | **N/A** |
| **SETD2** | **Q9BYW2** | **3.33** | **🡹** | **0.0002343** | **0.00682** | **+** | **761** | **N/A** | **0.26** | **883** | **2.83** | **3.4** |
| **HSPG2** | **P98160** | **3.31** | **🡹** | **1.20E-06** | **0.000292** |  | **761** | **0.26** | **0.53** | **883** | **2.6** | **N/A** |
| **STK10** | **O94804** | **3.32** | **🡹** | **3.28E-05** | **0.00274** |  | **761** | **0.13** | **0.26** | **883** | **2.6** | **N/A** |
| **D2HGDH** | **Q8N465** | **3.31** | **🡹** | **9.56E-05** | **0.00416** |  | **761** | **N/A** | **0.39** | **883** | **2.15** | **N/A** |
| **CSRNP1** | **Q96S65** | **3.34** | **🡹** | **3.00E-07** | **0.000141** |  | **761** | **0.53** | **0.26** | **883** | **1.25** |  |
| **NOL11** | **Q9H8H0** | **3.2** | **🡹** | **1.33E-05** | **0.00141** |  | **761** | **8.15** | **0.13** | **883** | **30.58** | **0.57** |
| **VPS72** | **Q15906** | **3.18** | **🡹** | **0.0001927** | **0.00925** |  | **761** | **6.04** | **N/A** | **883** | **27.75** | **N/A** |
| **CENPF** | **P49454** | **3.22** | **🡹** | **3.84E-05** | **0.00313** |  | **761** | **10.25** | **N/A** | **883** | **15.52** | **N/A** |
| **SUPT6H** | **Q7KZ85** | **3.2** | **🡹** | **0.0001709** | **0.00573** |  | **761** | **0.53** | **N/A** | **883** | **9.63** | **0.57** |
| **HNRNPF** | **P52597** | **3.28** | **🡹** | **1.00E-07** | **5.30E-05** |  | **761** | **0.13** | **0.13** | **883** | **8.83** | **4.19** |
| **NPM1** | **P06748** | **3.2** | **🡹** | **5.00E-07** | **0.000163** | **+** | **761** | **0.13** | **0.26** | **883** | **8.04** | **0.11** |
| **SND1** | **Q7KZF4** | **3.23** | **🡹** | **5.30E-06** | **0.00114** | **+** | **761** | **1.18** | **0.53** | **883** | **6.34** | **0.57** |
| **FN1** | **P02751** | **3.24** | **🡹** | **4.96E-05** | **0.00279** |  | **761** | **0.13** | **0.26** | **883** | **5.89** |  |
| **KIF3A** | **Q9Y496** | **3.2** | **🡹** | **0.0007625** | **0.0149** |  | **761** | **N/A** | **0.39** | **883** | **5.78** | **N/A** |
| **DUT** | **P33316** | **3.23** | **🡹** | **1.68E-05** | **0.00159** |  | **761** | **0.13** | **0.26** | **883** | **4.87** | **N/A** |
| **HADHA** | **P40939** | **3.26** | **🡹** | **0.0005712** | **0.0185** |  | **N/A** | **N/A** | **N/A** | **883** | **4.19** | **1.93** |
| **S100A9** | **P06702** | **3.22** | **🡹** | **2.02E-05** | **0.00208** |  | **761** | **6.44** | **N/A** | **883** | **4.19** | **N/A** |
| **CCDC64** | **Q6ZP65** | **3.23** | **🡹** | **9.53E-05** | **0.00597** |  | **761** | **0.13** | **0.26** | **883** | **3.85** | **N/A** |
| **MAP3K4** | **Q9Y6R4** | **3.26** | **🡹** | **2.00E-07** | **0.000165** |  | **761** | **0.26** | **0.92** | **883** | **3.17** | **2.04** |
| **ARHGEF16** | **Q5VV41** | **3.19** | **🡹** | **0.0006507** | **0.0138** |  | **761** | **0.26** | **0.26** | **883** | **2.38** | **N/A** |
| **FIS1** | **Q9Y3D6** | **3.25** | **🡹** | **1.22E-05** | **0.00178** |  | **761** | **0.13** | **0.39** | **883** | **2.27** | **N/A** |
| **PARP8** | **Q8N3A8** | **3.26** | **🡹** | **0.0001732** | **0.00867** |  | **761** | **0.53** | **0.66** | **883** | **2.15** | **N/A** |
| **PLEKHH3** | **Q7Z736** | **3.2** | **🡹** | **3.10E-06** | **0.00061** |  | **761** | **0.53** | **0.13** | **883** | **2.15** | **N/A** |
| **CTSD** | **P07339** | **3.2** | **🡹** | **0.0001885** | **0.00918** |  | **761** | **N/A** | **0.53** | **883** | **1.81** | **N/A** |
| **TUBGCP6** | **Q96RT7** | **3.19** | **🡹** | **0.0003185** | **0.00834** |  | **761** | **0.53** | **0.53** | **883** | **1.59** | **N/A** |
| **URI1** | **O94763** | **3.09** | **🡹** | **3.10E-06** | **0.000773** |  | **N/A** | **N/A** | **N/A** | **N/A** | **N/A** | **N/A** |
| **OS9** | **Q13438** | **3.14** | **🡹** | **5.00E-07** | **0.000163** |  | **761** | **0.92** | **N/A** | **883** | **13.27** | **1.7** |
| **SNX5** | **Q9Y5X3** | **3.16** | **🡹** | **4.91E-05** | **0.00521** |  | **761** | **1.18** | **0.26** | **883** | **10.76** | **1.59** |
| **PRDX1** | **Q06830** | **3.1** | **🡹** | **5.50E-06** | **0.00114** |  | **N/A** | **N/A** | **N/A** | **883** | **6.8** | **N/A** |
| **EIF4A2** | **Q14240** | **3.13** | **🡹** | **0.0003719** | **0.0132** | **+** | **761** | **1.58** | **N/A** | **883** | **5.66** | **0.57** |
| **TGM2** | **P21980** | **3.12** | **🡹** | **0.0008126** | **0.0154** |  | **761** | **1.45** | **N/A** | **883** | **5.66** | **N/A** |
| **WDR91** | **A4D1P6** | **3.08** | **🡹** | **0.0004513** | **0.0104** |  | **761** | **0.66** | **0.26** | **883** | **4.87** | **N/A** |
| **ARMCX5** | **Q6P1M9** | **3.17** | **🡹** | **0.0002851** | **0.0112** |  | **761** | **N/A** | **1.58** | **883** | **4.64** | **3.4** |
| **SFMBT1** | **Q9UHJ3** | **3.15** | **🡹** | **0.0006711** | **0.014** |  | **761** | **N/A** | **0.26** | **883** | **3.85** | **N/A** |
| **PRRT1** | **Q99946** | **3.15** | **🡹** | **0.0007601** | **0.0215** |  | **761** | **0.26** | **N/A** | **883** | **3.74** | **N/A** |
| **PRKCZ** | **Q05513** | **3.1** | **🡹** | **0.000914** | **0.0169** |  | **761** | **0.39** | **0.26** | **883** | **3.4** | **0.45** |
| **CHD3** | **Q12873** | **3.12** | **🡹** | **0.0001349** | **0.0117** |  | **761** | **0.13** | **0.53** | **883** | **2.38** | **1.47** |
| **MAN2C1** | **Q9NTJ4** | **3.1** | **🡹** | **0.0001438** | **0.00776** |  | **761** | **0.26** | **0.39** | **883** | **2.38** | **N/A** |
| **SCAP** | **Q12770** | **3.14** | **🡹** | **2.69E-05** | **0.00346** |  | **761** | **N/A** | **0.26** | **883** | **0.79** | **N/A** |
| **UBR5** | **O95071** | **3.05** | **🡹** | **0.0001669** | **0.0136** | **+** | **761** | **20.11** | **N/A** | **883** | **21.97** | **N/A** |
| **CLTC** | **Q00610** | **2.99** | **🡹** | **0.0002872** | **0.0218** | **+** | **761** | **10.51** | **0.13** | **883** | **21.06** | **N/A** |
| **MAF1** | **Q9H063** | **3.02** | **🡹** | **0.0006871** | **0.0399** |  | **761** | **9.33** | **N/A** | **883** | **18.8** | **N/A** |
| **PITPNC1** | **Q9UKF7** | **3.06** | **🡹** | **0.0009654** | **0.0664** |  | **761** | **8.54** | **0.13** | **883** | **7.93** | **N/A** |
| **RELA** | **Q04206** | **3.06** | **🡹** | **0.0001462** | **0.00525** |  | **761** | **0.66** | **N/A** | **883** | **7.93** | **5.32** |
| **HMGB2** | **P26583** | **3.03** | **🡹** | **6.20E-06** | **0.00114** |  | **761** | **0.26** | **0.92** | **883** | **7.02** | **N/A** |
| **KCNJ14** | **Q99712** | **3.02** | **🡹** | **0.0003158** | **0.0119** |  | **761** | **0.53** | **0.13** | **883** | **6.8** | **N/A** |
| **PSMC3** | **P17980** | **3.03** | **🡹** | **9.34E-05** | **0.00415** |  | **761** | **0.26** | **0.13** | **883** | **4.98** | **0.11** |
| **TXLNA** | **P40222** | **3** | **🡹** | **0.0002766** | **0.011** |  | **761** | **0.53** | **0.13** | **883** | **4.98** | **6.68** |
| **NEDD4L** | **Q96PU5** | **3.07** | **🡹** | **6.00E-06** | **0.00114** |  | **761** | **0.66** | **0.53** | **883** | **4.53** | **N/A** |
| **EIF2B4** | **Q9UI10** | **3.07** | **🡹** | **0.0002935** | **0.00784** |  | **761** | **0.13** | **N/A** | **883** | **4.19** | **0.34** |
| **AKAP13** | **Q12802** | **3** | **🡹** | **0.000705** | **0.0208** |  | **761** | **1.31** | **0.39** | **883** | **2.04** | **N/A** |
| **IER2** | **Q9BTL4** | **3.08** | **🡹** | **0.0004464** | **0.0149** |  | **761** | **0.39** | **N/A** | **883** | **0.91** | **N/A** |
| **C12orf32** | **Q9BSD3** | **2.98** | **🡹** | **6.74E-05** | **0.00492** |  | **N/A** | **N/A** | **N/A** | **N/A** | **N/A** | **N/A** |
| **PPP1R15B** | **Q5SWA1** | **2.9** | **🡹** | **0.0003671** | **0.0131** |  | **761** | **10.51** | **N/A** | **883** | **13.7** | **0.23** |
| **GPAA1** | **O43292** | **2.96** | **🡹** | **3.58E-05** | **0.00236** |  | **761** | **9.33** | **N/A** | **833** | **12.91** | **N/A** |
| **NECAP1** | **Q8NC96** | **2.96** | **🡹** | **0.0007449** | **0.0148** |  | **761** | **2.1** | **0.13** | **883** | **7.59** | **0.45** |
| **UBE2L3** | **P68036** | **2.98** | **🡹** | **0.0008172** | **0.0224** |  | **761** | **0.39** | **0.13** | **883** | **6.23** | **1.7** |
| **PTPRU** | **Q92729** | **2.91** | **🡹** | **0.0003592** | **0.00884** |  | **N/A** | **N/A** | **N/A** | **883** | **5.1** | **N/A** |
| **NT5C3L** | **Q969T7** | **2.93** | **🡹** | **3.60E-05** | **0.00402** |  | **761** | **0.92** | **N/A** | **883** | **4.87** | **0.79** |
| **PXN** | **P49023** | **2.94** | **🡹** | **8.60E-06** | **0.00146** |  | **761** | **N/A** | **0.26** | **883** | **4.76** | **0.23** |
| **LCP2** | **Q13094** | **2.94** | **🡹** | **2.22E-05** | **0.00216** |  | **761** | **0.13** | **0.39** | **883** | **4.42** | **N/A** |
| **GSDMD** | **P57764** | **2.96** | **🡹** | **0.0002986** | **0.0114** |  | **761** | **10.25** | **0.13** | **883** | **4.3** | **N/A** |
| **CENPT** | **Q96BT3** | **2.92** | **🡹** | **0.0008009** | **0.0223** |  | **761** | **0.13** | **1.05** | **883** | **3.17** | **N/A** |
| **LAMB3** | **Q13751** | **2.97** | **🡹** | **0.0001523** | **0.00538** |  | **761** | **10.91** | **N/A** | **883** | **2.94** | **N/A** |
| **NEDD9** | **Q14511** | **2.98** | **🡹** | **5.60E-06** | **0.000771** |  | **761** | **1.45** | **0.26** | **883** | **2.49** | **N/A** |
| **MAFF** | **Q9ULX9** | **2.93** | **🡹** | **0.0001953** | **0.00614** |  | **761** | **0.26** | **0.26** | **883** | **2.38** | **N/A** |
| **IGLON5** | **A6NGN9** | **2.96** | **🡹** | **0.0001324** | **0.00489** |  | **761** | **1.58** | **0.26** | **883** | **1.25** | **N/A** |
| **AKAP17A** | **Q02040** | **2.82** | **🡹** | **0.0003251** | **0.0084** |  | **761** | **0.26** | **1.05** | **N/A** | **N/A** | **N/A** |
| **PIEZO1** | **Q92508** | **2.9** | **🡹** | **6.11E-05** | **0.00466** |  | **761** | **0.26** | **1.05** | **N/A** | **N/A** | **N/A** |
| **RSPH10B/RSPH10B2** | **P0C881** | **2.83** | **🡹** | **7.65E-05** | **0.00721** |  | **761** | **0.92** | **0.26** | **N/A** | **N/A** | **N/A** |
| **TRIM24** | **O15164** | **2.85** | **🡹** | **4.95E-05** | **0.00279** | **+** | **761** | **0.66** | **0.26** | **N/A** | **N/A** | **N/A** |
| **LLGL2** | **Q6P1M3** | **2.85** | **🡹** | **8.48E-05** | **0.00385** |  | **761** | **3.68** | **N/A** | **883** | **12** | **0.11** |
| **FAM100B** | **Q8IYN6** | **2.82** | **🡹** | **0.0005774** | **0.0186** |  | **761** | **2.89** | **N/A** | **883** | **9.74** | **N/A** |
| **P4HB** | **P07237** | **2.84** | **🡹** | **0.000811** | **0.0224** |  | **761** | **2.1** | **N/A** | **883** | **8.49** | **N/A** |
| **SKIV2L** | **Q15477** | **2.9** | **🡹** | **0.0009415** | **0.0247** |  | **761** | **0.26** | **N/A** | **883** | **7.47** | **N/A** |
| **NRBP1** | **Q9UHY1** | **2.84** | **🡹** | **0.0003644** | **0.00891** |  | **761** | **0.13** | **N/A** | **883** | **7.02** | **N/A** |
| **EIF2S3** | **P41091** | **2.83** | **🡹** | **7.01E-05** | **0.00333** |  | **761** | **0.79** | **1.31** | **883** | **6.12** | **N/A** |
| **SLC3A2** | **P08195** | **2.82** | **🡹** | **0.0001568** | **0.0054** |  | **761** | **0.53** | **N/A** | **883** | **6.12** | **N/A** |
| **SORBS2** | **O94875** | **2.86** | **🡹** | **2.91E-05** | **0.00363** |  | **761** | **0.26** | **1.18** | **883** | **4.98** | **N/A** |
| **FNTB** | **P49356** | **2.87** | **🡹** | **0.000956** | **0.0248** |  | **761** | **N/A** | **0.26** | **883** | **4.64** | **4.53** |
| **PRKD2** | **Q9BZL6** | **2.85** | **🡹** | **5.80E-06** | **0.00114** |  | **761** | **0.39** | **0.13** | **883** | **3.62** | **1.25** |
| **POLI** | **Q9UNA4** | **2.89** | **🡹** | **0.0001263** | **0.00751** |  | **761** | **0.53** | **0.66** | **883** | **3.4** | **0.11** |
| **CCDC94** | **Q9BW85** | **2.89** | **🡹** | **7.59E-05** | **0.00721** |  | **761** | **0.13** | **0.13** | **883** | **2.38** | **N/A** |
| **FAM57B** | **Q71RH2** | **2.82** | **🡹** | **0.0003853** | **0.00929** |  | **761** | **2.63** | **N/A** | **883** | **2.38** | **N/A** |
| **FOSL2** | **P15408** | **2.89** | **🡹** | **0.0008574** | **0.0231** |  | **N/A** | **N/A** | **N/A** | **883** | **2.15** | **N/A** |
| **BANP** | **Q8N9N5** | **2.86** | **🡹** | **0.0001588** | **0.00807** |  | **761** | **0.26** | **1.05** | **883** | **1.47** | **N/A** |
| **PRRC2C** | **Q9Y520** | **2.76** | **🡹** | **1.70E-06** | **0.000515** |  | **761** | **8.15** | **N/A** | **N/A** | **N/A** | **N/A** |
| **ARNT** | **P27540** | **2.76** | **🡹** | **0.0009947** | **0.0253** | **+** | **761** | **6.83** | **N/A** | **883** | **17.1** | **0.79** |
| **APOH** | **P02749** | **2.76** | **🡹** | **5.42E-05** | **0.00561** |  | **761** | **7.23** | **0.13** | **883** | **5.21** | **N/A** |
| **STXBP3** | **O00186** | **2.75** | **🡹** | **9.90E-06** | **0.00156** |  | **761** | **0.39** | **N/A** | **883** | **4.64** | **3.28** |
| **HNRNPM** | **P52272** | **2.8** | **🡹** | **0.0002161** | **0.00971** |  | **761** | **0.39** | **N/A** | **883** | **3.85** | **0.91** |
| **ISM1** | **B1AKI9** | **2.74** | **🡹** | **0.0001502** | **0.00786** |  | **761** | **1.45** | **0.53** | **883** | **3.74** | **N/A** |
| **HOOK2** | **Q96ED9** | **2.8** | **🡹** | **0.0002559** | **0.0108** |  | **761** | **0.26** | **N/A** | **883** | **3.06** | **N/A** |
| **FCHO1** | **O14526** | **2.75** | **🡹** | **3.47E-05** | **0.00398** |  | **761** | **1.18** | **N/A** | **883** | **2.72** | **N/A** |
| **GABARAPL2** | **P60520** | **2.76** | **🡹** | **0.0006602** | **0.0389** |  | **761** | **0.13** | **1.05** | **883** | **2.49** | **0.45** |
| **SNAPIN** | **O95295** | **2.69** | **🡹** | **0.0001029** | **0.00438** |  | **761** | **6.04** | **N/A** | **883** | **31.82** | **0.23** |
| **NUP107** | **P57740** | **2.73** | **🡹** | **0.0007092** | **0.0208** |  | **761** | **3.42** | **N/A** | **883** | **15.29** | **0.45** |
| **ZC3H7A** | **Q8IWR0** | **2.73** | **🡹** | **5.02E-05** | **0.0126** |  | **761** | **3.81** | **N/A** | **883** | **8.27** | **N/A** |
| **SNX4** | **O95219** | **2.68** | **🡹** | **0.0009881** | **0.0538** |  | **761** | **0.13** | **0.39** | **883** | **7.93** | **1.59** |
| **RAB5C** | **P51148** | **2.68** | **🡹** | **0.0003821** | **0.0261** |  | **761** | **0.66** | **N/A** | **883** | **7.02** | **5.32** |
| **TOMM22** | **Q9NS69** | **2.71** | **🡹** | **0.0002753** | **0.011** |  | **761** | **0.13** | **0.39** | **883** | **6.23** | **0.11** |
| **MYO9B** | **Q13459** | **2.71** | **🡹** | **6.90E-06** | **0.00122** |  | **761** | **0.92** | **N/A** | **883** | **5.89** | **0.45** |
| **TBCB** | **Q99426** | **2.72** | **🡹** | **0.0001543** | **0.0054** |  | **761** | **1.58** | **N/A** | **883** | **5.78** | **N/A** |
| **CYB5B** | **O43169** | **2.71** | **🡹** | **0.0002712** | **0.011** |  | **761** | **0.26** | **0.92** | **883** | **3.51** | **3.06** |
| **RUNX3** | **Q13761** | **2.7** | **🡹** | **0.0002347** | **0.0103** |  | **761** | **0.13** | **0.13** | **883** | **2.83** | **N/A** |
| **TRIM8** | **Q9BZR9** | **2.73** | **🡹** | **0.0002616** | **0.00731** |  | **761** | **0.13** | **0.39** | **883** | **2.38** | **4.87** |
| **TNFSF13** | **O75888** | **2.71** | **🡹** | **2.47E-05** | **0.00519** |  | **997** | **N/A** | **0.7** | **1104** | **1.9** | **N/A** |
| **DENND1C** | **Q8IV53** | **2.71** | **🡹** | **1.00E-07** | **0.000188** |  | **N/A** | **N/A** | **N/A** | **883** | **1.47** | **N/A** |
| **KHDRBS1** | **Q07666** | **2.65** | **🡹** | **0.0004482** | **0.0292** |  | **761** | **0.66** | **0.13** | **883** | **7.02** | **1.02** |
| **GANAB** | **Q14697** | **2.62** | **🡹** | **0.0003435** | **0.00865** |  | **761** | **0.66** | **N/A** | **883** | **6.57** | **0.79** |
| **CC2D1A** | **Q6P1N0** | **2.64** | **🡹** | **0.0007217** | **0.0146** |  | **761** | **0.79** | **N/A** | **883** | **4.42** | **N/A** |
| **UBE2B** | **P63146** | **2.6** | **🡹** | **0.0001166** | **0.0103** |  | **761** | **N/A** | **0.39** | **883** | **4.08** | **0.45** |
| **XYLT1** | **Q86Y38** | **2.63** | **🡹** | **0.000617** | **0.0134** |  | **761** | **3.42** | **0.13** | **883** | **3.85** | **N/A** |
| **ACO1** | **P21399** | **2.61** | **🡹** | **5.40E-05** | **0.00284** |  | **761** | **0.39** | **0.26** | **883** | **2.49** | **N/A** |
| **PRRC2A** | **P48634** | **2.59** | **🡹** | **0.0003457** | **0.0126** |  | **761** | **0.13** | **N/A** | **N/A** | **N/A** | **N/A** |
| **ZNF835** | **Q9Y2P0** | **2.54** | **🡹** | **0.000208** | **0.00644** |  | **761** | **1.71** | **0.13** | **883** | **6.46** | **N/A** |
| **ARCN1** | **P48444** | **2.59** | **🡹** | **0.0002091** | **0.00952** |  | **761** | **N/A** | **0.92** | **883** | **3.51** | **1.47** |
| **PFAS** | **O15067** | **2.57** | **🡹** | **0.000712** | **0.0403** |  | **761** | **0.13** | **0.53** | **883** | **2.38** | **0.34** |
| **BGLAP** | **P02818** | **2.48** | **🡹** | **6.99E-05** | **0.00333** |  | **761** | **5.78** | **N/A** | **N/A** | **N/A** | **N/A** |
| **C12orf45** | **Q8N5I9** | **2.51** | **🡹** | **0.0001468** | **0.0125** |  | **N/A** | **N/A** | **N/A** | **N/A** | **N/A** | **N/A** |
| **SF3B4** | **Q15427** | **2.48** | **🡹** | **0.0004403** | **0.0148** |  | **761** | **3.55** | **N/A** | **883** | **31.03** | **N/A** |
| **POGZ** | **Q7Z3K3** | **2.48** | **🡹** | **9.30E-06** | **0.00152** |  | **761** | **6.18** | **N/A** | **883** | **24.24** | **0.11** |
| **FIGNL1** | **Q6PIW4** | **2.5** | **🡹** | **0.0004333** | **0.0147** |  | **761** | **0.53** | **0.13** | **883** | **10.31** | **N/A** |
| **RPS6KB2** | **Q9UBS0** | **2.53** | **🡹** | **0.000241** | **0.024** |  | **761** | **4.99** | **N/A** | **883** | **8.83** | **N/A** |
| **ACTR3** | **P61158** | **2.5** | **🡹** | **0.000228** | **0.0266** |  | **761** | **0.26** | **N/A** | **883** | **7.25** | **N/A** |
| **EZR** | **P15311** | **2.52** | **🡹** | **1.08E-05** | **0.00675** | **+** | **761** | **N/A** | **0.79** | **883** | **4.08** | **0.11** |
| **PPIP5K1** | **Q6PFW1** | **2.47** | **🡹** | **0.0001923** | **0.0151** |  | **761** | **0.26** | **0.26** | **883** | **4.08** | **N/A** |
| **CTSZ** | **Q9UBR2** | **2.48** | **🡹** | **6.41E-05** | **0.00323** |  | **761** | **6.04** | **0.13** | **883** | **3.96** | **N/A** |
| **ANGPTL2** | **Q9UKU9** | **2.5** | **🡹** | **0.0001931** | **0.00614** |  | **761** | **0.66** | **0.13** | **883** | **3.74** | **N/A** |
| **TWF2** | **Q6IBS0** | **2.52** | **🡹** | **0.0002427** | **0.0104** |  | **761** | **N/A** | **0.26** | **883** | **3.06** | **N/A** |
| **QARS** | **P07814** | **2.47** | **🡹** | **0.0008016** | **0.0436** |  | **761** | **N/A** | **0.26** | **883** | **2.94** | **2.94** |
| **ZFYVE28** | **Q9HCC9** | **2.53** | **🡹** | **0.0007401** | **0.0605** |  | **761** | **0.39** | **0.53** | **883** | **2.27** | **N/A** |
| **XBP1** | **P17861** | **2.51** | **🡹** | **0.0006022** | **0.019** |  | **761** | **0.66** | **0.39** | **883** | **1.93** | **N/A** |
| **CPE** | **P16870** | **2.48** | **🡹** | **0.0004505** | **0.0459** |  | **761** | **0.39** | **0.79** | **883** | **1.47** | **N/A** |
| **TRADD** | **Q15628** | **2.52** | **🡹** | **0.0001034** | **0.00933** |  | **761** | **0.13** | **1.05** | **883** | **0.91** | **N/A** |
| **SPCS2** | **Q15005** | **2.52** | **🡹** | **6.11E-05** | **0.00316** |  | **930** | **3.3** | **N/A** |  | **N/A** | **N/A** |
| **PARD3** | **Q8TEW0** | **2.41** | **🡹** | **8.97E-05** | **0.00403** |  | **761** | **0.79** | **N/A** | **883** | **8.38** | **0.34** |
| **SDCBP** | **O00560** | **2.47** | **🡹** | **0.0006888** | **0.0584** |  | **761** | **10.38** | **N/A** | **883** | **7.47** | **0.11** |
| **MAGED2** | **Q9UNF1** | **2.46** | **🡹** | **2.74E-05** | **0.0096** |  | **761** | **0.26** | **0.66** | **883** | **6.46** | **N/A** |
| **ARHGAP1** | **Q07960** | **2.42** | **🡹** | **0.0002695** | **0.011** |  | **761** | **0.53** | **0.13** | **883** | **4.3** | **2.83** |
| **PREPL** | **Q4J6C6** | **2.42** | **🡹** | **0.0009051** | **0.0474** |  | **761** | **0.26** | **N/A** | **883** | **4.19** | **0.23** |
| **DNAJC10** | **Q8IXB1** | **2.45** | **🡹** | **9.10E-06** | **0.00331** |  | **761** | **0.13** | **N/A** | **883** | **3.51** | **N/A** |
| **CYR61** | **O00622** | **2.46** | **🡹** | **0.0001738** | **0.00575** |  | **761** | **0.26** | **N/A** | **883** | **1.81** | **N/A** |
| **SSR2** | **P43308** | **2.39** | **🡹** | **0.000785** | **0.022** |  | **761** | **5.78** | **N/A** | **883** | **17.67** | **N/A** |
| **CDK13** | **Q14004** | **2.37** | **🡹** | **7.32E-05** | **0.00721** |  | **761** | **1.18** | **N/A** | **883** | **7.25** | **2.38** |
| **COX7B** | **P24311** | **2.38** | **🡹** | **0.0001961** | **0.00614** |  | **761** | **0.13** | **1.05** | **883** | **7.13** | **N/A** |
| **ZFP2** | **Q6ZN57** | **2.37** | **🡹** | **0.0007277** | **0.0146** |  | **761** | **0.39** | **0.26** | **883** | **3.85** | **N/A** |
| **TMEM131** | **Q92545** | **2.39** | **🡹** | **0.0002211** | **0.00653** |  | **761** | **0.53** | **N/A** | **883** | **3.74** | **0.68** |
| **AMT** | **P48728** | **2.37** | **🡹** | **1.01E-05** | **0.00675** |  | **761** | **N/A** | **0.26** | **883** | **3.62** | **N/A** |
| **CMTM3** | **Q96MX0** | **2.36** | **🡹** | **0.0006692** | **0.0204** |  | **761** | **0.26** | **1.05** | **883** | **3.06** | **N/A** |
| **RBM25** | **P49756** | **2.4** | **🡹** | **0.0003165** | **0.0235** |  | **761** | **N/A** | **0.13** | **883** | **2.49** | **0.91** |
| **SUMO1P3** | **P63165** | **2.35** | **🡹** | **0.0007396** | **0.0213** |  | **N/A** | **N/A** | **N/A** | **N/A** | **N/A** | **N/A** |
| **PSME4** | **Q14997** | **2.31** | **🡹** | **0.0009245** | **0.0598** |  | **761** | **0.66** | **N/A** | **883** | **13.14** | **N/A** |
| **PDXK** | **O00764** | **2.3** | **🡹** | **0.0007447** | **0.0473** |  | **761** | **0.39** | **N/A** | **883** | **9.97** | **N/A** |
| **SUPT5H** | **O00267** | **2.33** | **🡹** | **8.48E-05** | **0.0151** |  | **761** | **1.58** | **N/A** | **883** | **9.51** | **1.25** |
| **PSMC4** | **P43686** | **2.32** | **🡹** | **0.0002771** | **0.00753** |  | **761** | **2.1** | **N/A** | **883** | **8.61** | **N/A** |
| **CP** | **P00450** | **2.33** | **🡹** | **9.50E-05** | **0.00416** |  | **761** | **1.58** | **N/A** | **883** | **6.23** | **N/A** |
| **TMOD3** | **Q9NYL9** | **2.33** | **🡹** | **6.82E-05** | **0.0135** |  | **761** | **0.66** | **0.39** | **883** | **5.1** | **1.81** |
| **ACTR1B** | **P42025** | **2.34** | **🡹** | **0.0006853** | **0.0207** |  | **761** | **0.53** | **N/A** | **883** | **3.4** | **1.7** |
| **SFTPA1** | **Q8IWL2** | **2.34** | **🡹** | **0.0002401** | **0.00684** |  | **761** | **1.97** | **0.26** | **883** | **2.38** | **N/A** |
| **GPC3** | **P51654** | **2.35** | **🡹** | **0.0009415** | **0.017** | **+** | **761** | **0.39** | **1.05** | **883** | **2.15** | **N/A** |
| **BOD1L1** | **Q8NFC6** | **2.29** | **🡹** | **0.0001666** | **0.0136** |  | **761** | **0.26** | **0.79** | **N/A** | **N/A** | **N/A** |
| **PKM** | **P14618** | **2.29** | **🡹** | **0.0002381** | **0.00683** |  | **761** | **0.39** | **0.39** | **N/A** | **N/A** | **N/A** |
| **CDC25B** | **P30305** | **2.25** | **🡹** | **0.0009127** | **0.0169** |  | **761** | **0.92** | **0.13** | **883** | **10.53** | **N/A** |
| **TAX1BP1** | **Q86VP1** | **2.25** | **🡹** | **0.0006904** | **0.0467** |  | **761** | **1.31** | **N/A** | **883** | **9.51** | **1.25** |
| **PIK3R2** | **O00459** | **2.28** | **🡹** | **0.0002183** | **0.00653** |  | **761** | **0.66** | **N/A** | **883** | **6.57** | **N/A** |
| **SRA1** | **Q9H7N4** | **2.25** | **🡹** | **0.0009714** | **0.05** |  | **761** | **0.26** | **0.39** | **883** | **3.96** | **N/A** |
| **COL1A1** | **P02452** | **2.28** | **🡹** | **0.0003866** | **0.00929** | **+** | **761** | **6.96** | **N/A** | **883** | **3.85** | **N/A** |
| **HSPB1** | **P04792** | **2.29** | **🡹** | **0.0007619** | **0.0475** |  | **761** | **0.26** | **0.13** | **883** | **3.28** | **N/A** |
| **C12orf35** | **Q9HCM1** | **2.23** | **🡹** | **0.0002809** | **0.0255** |  | **N/A** | **N/A** | **N/A** | **N/A** | **N/A** | **N/A** |
| **U2AF1** | **Q01081** | **2.21** | **🡹** | **0.0007092** | **0.0403** | **+** | **761** | **0.66** | **N/A** | **883** | **9.85** | **N/A** |
| **ABCE1** | **P61221** | **2.24** | **🡹** | **0.000172** | **0.00573** |  | **761** | **0.79** | **0.39** | **883** | **9.06** | **0.45** |
| **BRPF1** | **P55201** | **2.2** | **🡹** | **0.0001698** | **0.0136** |  | **761** | **1.18** | **N/A** | **883** | **7.59** | **0.91** |
| **PLCG1** | **P19174** | **2.21** | **🡹** | **0.0003707** | **0.0261** | **+** | **761** | **1.71** | **N/A** | **883** | **6.91** | **N/A** |
| **LRRC47** | **Q8N1G4** | **2.23** | **🡹** | **0.0001999** | **0.00934** |  | **761** | **0.13** | **0.26** | **883** | **5.1** | **2.83** |
| **BCAM** | **P50895** | **2.24** | **🡹** | **1.78E-05** | **0.00503** |  | **761** | **0.92** | **0.13** | **883** | **4.42** | **N/A** |
| **FAM134A** | **Q8NC44** | **2.24** | **🡹** | **4.24E-05** | **0.00461** |  | **761** | **0.26** | **0.13** | **883** | **3.96** | **3.4** |
| **ZFP14** | **Q9HCL3** | **2.22** | **🡹** | **5.50E-06** | **0.00331** |  | **761** | **1.58** | **N/A** | **883** | **3.85** | **0.68** |
| **KDR** | **P35968** | **2.2** | **🡹** | **0.0006477** | **0.0486** | **+** | **761** | **0.66** | **0.26** | **883** | **3.51** | **N/A** |
| **PTPN7** | **P35236** | **2.24** | **🡹** | **5.76E-05** | **0.0127** |  | **761** | **9.46** | **0.13** | **883** | **1.81** | **N/A** |
| **SCG5** | **P05408** | **2.22** | **🡹** | **9.42E-05** | **0.0155** |  | **761** | **0.26** | **0.39** | **883** | **0.91** | **N/A** |
| **BRSK1** | **Q8TDC3** | **2.19** | **🡹** | **0.0009837** | **0.0175** |  | **761** | **2.37** | **0.13** | **N/A** | **N/A** | **N/A** |
| **CEP70** | **Q8NHQ1** | **2.19** | **🡹** | **0.0005386** | **0.0332** |  | **761** | **1.05** | **N/A** | **883** | **11.1** | **0.11** |
| **NOP58** | **Q9Y2X3** | **2.18** | **🡹** | **0.0003724** | **0.0261** |  | **761** | **N/A** | **0.26** | **883** | **7.59** | **N/A** |
| **ZNF317** | **Q96PQ6** | **2.17** | **🡹** | **8.95E-05** | **0.0134** |  | **761** | **0.53** | **N/A** | **883** | **5.89** | **2.72** |
| **PTPN23** | **Q9H3S7** | **2.17** | **🡹** | **0.0003841** | **0.0261** |  | **761** | **N/A** | **0.26** | **883** | **3.4** | **N/A** |
| **FHL2** | **Q14192** | **2.16** | **🡹** | **0.0007906** | **0.0153** |  | **761** | **0.39** | **N/A** | **883** | **3.28** | **N/A** |
| **CISH** | **Q9NSE2** | **2.18** | **🡹** | **0.0002339** | **0.0266** |  | **761** | **N/A** | **0.26** | **883** | **2.49** | **N/A** |
| **SUSD2** | **Q9UGT4** | **2.18** | **🡹** | **0.0003481** | **0.00867** |  | **761** | **0.53** | **N/A** | **883** | **1.25** | **N/A** |
| **PPP2R5D** | **Q14738** | **2.11** | **🡹** | **5.99E-05** | **0.0117** |  | **761** | **1.58** | **0.13** | **883** | **12.34** | **1.36** |
| **JMJD8** | **Q96S16** | **2.14** | **🡹** | **0.0008949** | **0.0589** |  | **761** | **4.6** | **0.13** | **883** | **9.06** | **0.11** |
| **TAF4B** | **Q92750** | **2.14** | **🡹** | **0.0007546** | **0.0421** |  | **761** | **1.45** | **0.39** | **883** | **6.91** | **N/A** |
| **ATN1** | **P54259** | **2.13** | **🡹** | **0.000493** | **0.043** |  | **761** | **2.23** | **0.13** | **883** | **6.68** | **1.47** |
| **SAMD14** | **Q8IZD0** | **2.13** | **🡹** | **0.0001882** | **0.0218** |  | **761** | **6.83** | **N/A** | **883** | **4.87** | **N/A** |
| **NONO** | **Q15233** | **2.07** | **🡹** | **4.00E-07** | **0.000223** | **+** | **761** | **0.13** | **1.05** | **883** | **10.31** | **0.45** |
| **EEF1D** | **P29692** | **2.1** | **🡹** | **0.0006615** | **0.0455** |  | **761** | **10.12** | **0.13** | **883** | **5.78** | **N/A** |
| **LZTR1** | **Q8N653** | **2.09** | **🡹** | **0.0008168** | **0.0154** |  | **761** | **0.13** | **0.13** | **883** | **5.44** | **1.7** |
| **PCDH7** | **O60245** | **2.09** | **🡹** | **0.0002525** | **0.0242** |  | **761** | **0.26** | **0.66** | **883** | **3.17** | **N/A** |
| **STMN4** | **Q9H169** | **2.09** | **🡹** | **8.90E-06** | **0.00103** |  | **761** | **0.66** | **4.99** | **883** | **0.79** | **N/A** |
| **KAT7** | **O95251** | **2.06** | **🡹** | **0.0008425** | **0.0452** |  | **N/A** | **N/A** | **N/A** | **N/A** | **N/A** | **N/A** |
| **PARP2** | **Q9UGN5** | **2.06** | **🡹** | **0.0004762** | **0.0108** |  | **761** | **0.39** | **N/A** | **883** | **11.66** | **0.23** |
| **YES1** | **P07947** | **2.03** | **🡹** | **3.64E-05** | **0.00236** |  | **761** | **0.79** | **0.39** | **883** | **10.99** | **0.11** |
| **LRWD1** | **Q9UFC0** | **2.06** | **🡹** | **0.0007909** | **0.0539** |  | **761** | **0.13** | **0.39** | **883** | **6.23** | **N/A** |
| **GALE** | **Q14376** | **2.06** | **🡹** | **0.00013** | **0.00488** |  | **761** | **N/A** | **0.39** | **883** | **4.19** | **N/A** |
| **DOCK4** | **Q8N1I0** | **2.05** | **🡹** | **0.0006621** | **0.014** |  | **761** | **0.53** | **0.53** | **883** | **3.85** | **N/A** |
| **MAPK8IP2** | **Q13387** | **2.05** | **🡹** | **0.0005397** | **0.0332** |  | **761** | **0.39** | **0.53** | **883** | **2.72** | **N/A** |
| **TXLNG2P** | **Q9BZA5** | **1.98** | **🡹** | **0.0005411** | **0.0177** |  | **N/A** | **N/A** | **N/A** | **N/A** | **N/A** | **N/A** |
| **SOX4** | **Q06945** | **1.99** | **🡹** | **0.0008927** | **0.0473** |  | **761** | **1.97** | **N/A** | **883** | **8.04** | **N/A** |
| **DAXX** | **Q9UER7** | **2.01** | **🡹** | **2.72E-05** | **0.00208** | **+** | **761** | **0.26** | **0.13** | **883** | **6.23** | **0.23** |
| **TGFB1I1** | **O43294** | **2.01** | **🡹** | **9.80E-06** | **0.00109** |  | **761** | **2.89** | **N/A** | **883** | **2.83** | **N/A** |
| **COPZ1** | **P61923** | **1.95** | **🡹** | **0.0007996** | **0.0436** |  | **761** | **0.13** | **N/A** | **883** | **5.78** | **1.13** |
| **B4GALT2** | **O60909** | **1.9** | **🡹** | **0.0009736** | **0.0174** |  | **761** | **0.26** | **0.13** | **883** | **6.91** | **N/A** |
| **OLFM2** | **O95897** | **1.9** | **🡹** | **0.0001308** | **0.0185** |  | **761** | **0.66** | **N/A** | **883** | **6.57** | **N/A** |
| **NBPF15** | **Q8N660** | **1.88** | **🡹** | **0.0004194** | **0.00991** |  |  | **N/A** | **N/A** |  | **N/A** | **N/A** |
| **SIAH1** | **Q8IUQ4** | **1.85** | **🡹** | **0.0001063** | **0.00439** |  | **761** | **1.71** | **0.66** | **883** | **6** | **1.7** |
| **TP53I11** | **O14683** | **1.84** | **🡹** | **0.0002638** | **0.00732** |  | **761** | **0.66** | **0.26** | **883** | **5.66** | **0.23** |
| **AURKAIP1** | **Q9NWT8** | **1.84** | **🡹** | **0.0001421** | **0.0185** |  | **761** | **0.26** | **0.26** | **883** | **2.27** | **N/A** |
| **BOK** | **Q9UMX3** | **1.86** | **🡹** | **0.0002792** | **0.0255** |  | **761** | **N/A** | **0.26** | **883** | **2.15** | **N/A** |
| **VTN** | **P04004** | **1.85** | **🡹** | **0.0004375** | **0.0102** |  | **761** | **3.68** | **0.13** | **883** | **0.91** | **N/A** |
| **MRPS11** | **P82912** | **1.82** | **🡹** | **1.63E-05** | **0.00159** |  | **761** | **1.31** | **0.39** | **883** | **5.21** | **N/A** |
| **ITIH2** | **P19823** | **1.8** | **🡹** | **0.0002501** | **0.00708** |  | **761** | **3.02** | **N/A** | **883** | **0.91** | **N/A** |
| **DDX1** | **Q92499** | **1.79** | **🡹** | **0.0001092** | **0.00442** |  | **761** | **0.39** | **0.13** | **883** | **8.61** | **2.15** |
| **RTKN** | **Q9BST9** | **1.74** | **🡹** | **0.0002364** | **0.00683** |  | **761** | **0.26** | **N/A** | **883** | **7.25** | **0.34** |
| **S100A16** | **Q96FQ6** | **1.73** | **🡹** | **0.0004096** | **0.00979** |  | **761** | **6.04** | **N/A** | **883** | **11.89** | **N/A** |
| **PRPF8** | **Q6P2Q9** | **1.71** | **🡹** | **0.0004161** | **0.0276** |  | **761** | **0.13** | **0.39** | **883** | **3.74** | **3.74** |
| **HNRNPH1** | **P31943** | **1.68** | **🡹** | **0.0001057** | **0.00439** |  | **761** | **0.39** | **0.39** | **883** | **6** | **0.91** |
| **UPF1** | **Q92900** | **1.68** | **🡹** | **0.0008137** | **0.0154** |  | **761** | **1.05** | **N/A** | **883** | **5.21** | **2.04** |
| **SSBP2** | **P81877** | **1.71** | **🡹** | **8.93E-05** | **0.0134** |  | **761** | **0.13** | **0.66** | **883** | **4.76** | **N/A** |
| **MAPKAPK2** | **P49137** | **1.65** | **🡹** | **0.0004373** | **0.0102** |  | **761** | **9.46** | **N/A** | **883** | **10.87** | **N/A** |
| **CLCN2** | **P51788** | **1.66** | **🡹** | **0.0001431** | **0.0185** |  | **761** | **1.71** | **N/A** | **883** | **8.38** | **N/A** |
| **PSMD6** | **Q15008** | **1.66** | **🡹** | **5.14E-05** | **0.0109** |  | **761** | **N/A** | **0.53** | **883** | **4.98** | **1.25** |
| **SCO1** | **O75880** | **1.67** | **🡹** | **5.71E-05** | **0.0127** |  | **761** | **0.13** | **0.92** | **883** | **4.98** | **12.46** |
| **PISD** | **Q9UG56** | **1.66** | **🡹** | **0.0005888** | **0.0128** |  | **761** | **0.66** | **0.39** | **883** | **4.53** | **N/A** |
| **NDC80** | **O14777** | **1.63** | **🡹** | **0.0001186** | **0.0178** |  | **761** | **0.79** | **0.39** | **883** | **12.46** | **N/A** |
| **RPL17** | **P18621** | **1.63** | **🡹** | **3.01E-05** | **0.00226** |  | **761** | **0.53** | **0.53** | **883** | **3.85** | **N/A** |
| **C1orf43** | **Q9BWL3** | **1.61** | **🡹** | **3.29E-05** | **0.00234** |  | **N/A** | **N/A** | **N/A** | **N/A** | **N/A** | **N/A** |
| **PCDH9** | **Q9HC56** | **1.62** | **🡹** | **1.00E-07** | **0.000165** |  | **761** | **0.26** | **1.31** | **883** | **0.57** | **N/A** |
| **WDR1** | **O75083** | **1.58** | **🡹** | **3.61E-05** | **0.00236** |  | **761** | **0.26** | **0.92** | **883** | **3.4** | **1.93** |
| **OLFML3** | **Q9NRN5** | **1.59** | **🡹** | **0.000864** | **0.0162** |  | **761** | **0.39** | **0.13** | **883** | **0.79** | **N/A** |
| **SSFA2** | **P28290** | **1.57** | **🡹** | **0.0005792** | **0.0127** |  | **761** | **0.13** | **N/A** | **883** | **3.74** | **N/A** |
| **PHF3** | **Q92576** | **1.53** | **🡹** | **0.0003395** | **0.00865** |  | **761** | **0.92** | **0.26** | **883** | **4.87** | **2.04** |
| **LENG8** | **Q96PV6** | **1.53** | **🡹** | **0.0003127** | **0.0317** |  | **761** | **1.71** | **0.13** | **883** | **3.96** | **N/A** |
| **INPP5E** | **Q10713** | **1.5** | **🡹** | **0.0008083** | **0.0154** |  | **761** | **0.39** | **0.39** | **883** | **3.85** | **0.34** |
| **SNAI2** | **O43623** | **-1.50** | **🡻** | **5.43E-05** | **0.00284** |  | **761** | **7.88** | **0.13** | **883** | **2.72** | **N/A** |
| **ATP5O** | **P48047** | **-1.51** | **🡻** | **0.0002876** | **0.00777** |  | **761** | **0.13** | **N/A** | **883** | **6.91** | **0.23** |
| **TNFAIP8L2** | **Q6P589** | **-1.51** | **🡻** | **0.0008285** | **0.0156** |  | **761** | **6.04** | **N/A** | **883** | **1.93** | **N/A** |
| **HERC2P4** | **O95714** | **-1.52** | **🡻** | **0.0005318** | **0.0118** |  | **N/A** | **N/A** | **N/A** | **N/A** | **N/A** | **N/A** |
| **PAFAH1B3** | **Q15102** | **-1.52** | **🡻** | **3.99E-05** | **0.00236** |  | **761** | **0.53** | **0.13** | **883** | **3.74** | **N/A** |
| **VPS35** | **Q96QK1** | **-1.53** | **🡻** | **0.0002536** | **0.00713** |  | **761** | **1.58** | **0.53** | **883** | **7.25** | **1.13** |
| **TRO** | **Q12816** | **-1.53** | **🡻** | **0.0005015** | **0.0112** |  | **761** | **0.26** | **0.53** | **883** | **4.53** | **N/A** |
| **NCKAP1L** | **P55160** | **-1.53** | **🡻** | **4.83E-05** | **0.00376** |  | **761** | **N/A** | **0.13** | **883** | **3.17** | **N/A** |
| **BABAM1** | **Q9NWV8** | **-1.54** | **🡻** | **0.0002152** | **0.00653** |  | **761** | **0.79** | **N/A** | **N/A** | **N/A** | **N/A** |
| **CHGA** | **P10645** | **-1.54** | **🡻** | **0.0007243** | **0.0146** |  | **761** | **0.26** | **0.39** | **N/A** | **N/A** | **N/A** |
| **AKR1C4** | **P17516** | **-1.54** | **🡻** | **6.20E-06** | **0.000906** |  | **761** | **3.02** | **0.13** | **883** | **2.49** | **N/A** |
| **ABCF3** | **Q9NUQ8** | **-1.55** | **🡻** | **8.17E-05** | **0.0134** |  | **761** | **1.58** | **N/A** | **883** | **9.29** | **2.27** |
| **ATP13A1** | **Q9HD20** | **-1.56** | **🡻** | **0.000975** | **0.0174** |  | **761** | **1.05** | **N/A** | **883** | **4.76** | **N/A** |
| **EIF3A** | **P56537** | **-1.57** | **🡻** | **0.000918** | **0.0169** |  | **761** | **0.26** | **0.53** | **883** | **2.72** | **3.17** |
| **RIC3** | **Q7Z5B4** | **-1.58** | **🡻** | **0.0009443** | **0.017** |  | **997** | **0.1** | **0.2** | **1104** | **4.98** | **N/A** |
| **DDX20** | **Q9UHI6** | **-1.60** | **🡻** | **0.0003216** | **0.00836** |  | **1143** | **0.17** | **N/A** | **883** | **6.46** | **1.25** |
| **EPC1** | **Q9H2F5** | **-1.60** | **🡻** | **0.0001632** | **0.00553** |  | **761** | **0.53** | **N/A** | **883** | **2.27** | **2.83** |
| **SYNE2** | **Q8WXH0** | **-1.61** | **🡻** | **0.000255** | **0.0242** |  | **761** | **0.26** | **0.13** | **883** | **3.85** | **N/A** |
| **LOC100132832/PMS2P5** | **A8MQ11** | **-1.62** | **🡻** | **0.0002106** | **0.00645** |  | **761** | **0.66** | **0.13** | **N/A** | **N/A** | **N/A** |
| **TTC3** | **P53804** | **-1.62** | **🡻** | **0.0006265** | **0.0134** |  | **761** | **0.92** | **N/A** | **883** | **5.21** | **N/A** |
| **NXPH3** | **O95157** | **-1.62** | **🡻** | **0.0001145** | **0.00451** |  | **761** | **6.57** | **0.13** | **883** | **2.83** | **N/A** |
| **CELSR1** | **Q9NYQ6** | **-1.62** | **🡻** | **0.0001138** | **0.00451** |  | **761** | **0.39** | **0.39** | **883** | **1.93** | **N/A** |
| **RER1** | **O15258** | **-1.65** | **🡻** | **0.0007132** | **0.0208** |  | **761** | **0.53** | **0.26** | **883** | **3.17** | **0.34** |
| **R3HDM2** | **Q9Y2K5** | **-1.66** | **🡻** | **2.88E-05** | **0.00254** |  | **761** | **0.26** | **N/A** | **883** | **6.23** | **N/A** |
| **SCARB2** | **Q14108** | **-1.69** | **🡻** | **1.73E-05** | **0.00159** |  | **761** | **1.58** | **0.26** | **883** | **7.02** | **0.57** |
| **NSDHL** | **Q15738** | **-1.69** | **🡻** | **0.0003509** | **0.00868** |  | **761** | **0.13** | **1.05** | **883** | **6** | **N/A** |
| **POLR2B** | **P30876** | **-1.71** | **🡻** | **1.82E-05** | **0.00163** |  | **761** | **0.79** | **0.13** | **883** | **8.38** | **1.7** |
| **CLCN6** | **P51797** | **-1.71** | **🡻** | **0.0002197** | **0.00653** |  | **761** | **N/A** | **0.39** | **883** | **1.59** | **3.4** |
| **FBXO6** | **Q9NRD1** | **-1.73** | **🡻** | **0.0003291** | **0.0286** |  | **761** | **N/A** | **0.26** | **883** | **4.3** | **N/A** |
| **EIF3D** | **O15371** | **-1.76** | **🡻** | **0.0004827** | **0.0109** |  | **761** | **0.26** | **0.53** | **883** | **3.62** | **1.81** |
| **PIK3CD** | **O00329** | **-1.76** | **🡻** | **0.0002111** | **0.0216** |  | **761** | **0.13** | **0.39** | **883** | **2.38** | **N/A** |
| **ADH5** | **P11766** | **-1.80** | **🡻** | **0.0001006** | **0.0157** |  | **761** | **0.39** | **0.26** | **883** | **4.64** | **1.36** |
| **EML3** | **Q32P44** | **-1.80** | **🡻** | **0.0009973** | **0.0538** |  | **761** | **0.66** | **N/A** | **883** | **2.15** | **0.57** |
| **RASGRP2** | **Q7LDG7** | **-1.81** | **🡻** | **7.00E-07** | **0.000532** |  | **761** | **0.39** | **0.13** | **883** | **1.13** | **N/A** |
| **HLA-A** | **P01891** | **-1.82** | **🡻** | **0.0006341** | **0.0485** | **+** | **761** | **0.26** | **N/A** | **883** | **4.76** | **N/A** |
| **ARAP2** | **Q8WZ64** | **-1.82** | **🡻** | **0.0001325** | **0.00489** |  | **761** | **N/A** | **0.53** | **883** | **3.51** | **N/A** |
| **CACNB3** | **P54284** | **-1.83** | **🡻** | **2.60E-06** | **0.000537** |  | **761** | **0.13** | **0.13** | **883** | **4.76** | **N/A** |
| **MAPK8IP3** | **Q9UPT6** | **-1.84** | **🡻** | **0.0001747** | **0.0213** |  | **761** | **5.12** | **N/A** | **883** | **4.64** | **N/A** |
| **MLLT3** | **P42568** | **-1.86** | **🡻** | **0.0001426** | **0.00517** | **+** | **761** | **0.92** | **1.18** | **883** | **3.62** | **N/A** |
| **COL6A3** | **P12111** | **-1.86** | **🡻** | **0.00075** | **0.0148** |  | **761** | **N/A** | **0.26** | **883** | **3.51** | **N/A** |
| **PGK1** | **P00558** | **-1.87** | **🡻** | **8.21E-05** | **0.00381** |  | **761** | **0.13** | **1.18** | **883** | **10.42** | **N/A** |
| **NDEL1** | **Q9GZM8** | **-1.87** | **🡻** | **1.90E-06** | **0.00242** |  | **761** | **0.13** | **0.53** | **883** | **3.17** | **7.13** |
| **RPS17/RPS17L** | **P08708** | **-1.87** | **🡻** | **9.52E-05** | **0.0155** |  | **1143** | **0.09** | **N/A** |  | **N/A** | **N/A** |
| **CDK7** | **P50613** | **-1.88** | **🡻** | **0.0006203** | **0.0192** |  | **761** | **0.39** | **0.79** | **883** | **6.12** | **1.02** |
| **LCP1** | **P13796** | **-1.88** | **🡻** | **6.83E-05** | **0.00332** | **+** | **997** | **N/A** | **1.5** | **1104** | **3.08** | **N/A** |
| **ZNF300** | **Q96RE9** | **-1.89** | **🡻** | **1.97E-05** | **0.00166** |  | **761** | **0.13** | **0.53** | **883** | **10.31** | **N/A** |
| **CHKB** | **Q9Y259** | **-1.89** | **🡻** | **0.0005705** | **0.0455** |  | **761** | **0.39** | **0.53** | **883** | **3.17** | **0.34** |
| **ATG13** | **O75143** | **-1.94** | **🡻** | **6.71E-05** | **0.0033** |  | **761** | **0.53** | **0.13** | **N/A** | **N/A** | **N/A** |
| **NOL8** | **Q76FK4** | **-1.94** | **🡻** | **0.0006843** | **0.0494** |  | **N/A** | **N/A** | **N/A** | **883** | **5.21** | **2.04** |
| **AGAP2** | **Q99490** | **-1.98** | **🡻** | **5.43E-05** | **0.00284** |  | **761** | **1.05** | **N/A** | **883** | **4.08** | **N/A** |
| **MZF1** | **P28698** | **-1.99** | **🡻** | **0.0002191** | **0.00653** |  | **761** | **1.71** | **0.39** | **883** | **3.4** | **N/A** |
| **PDCD6IP** | **Q8WUM4** | **-2.00** | **🡻** | **0.0003091** | **0.00819** |  | **761** | **0.66** | **N/A** | **883** | **5.32** | **7.59** |
| **ALDH16A1** | **Q8IZ83** | **-2.00** | **🡻** | **0.0003607** | **0.0339** |  | **761** | **1.45** | **0.13** | **883** | **3.62** | **N/A** |
| **PABPC1** | **P11940** | **-2.02** | **🡻** | **0.0007967** | **0.0153** |  | **761** | **20.63** | **N/A** | **883** | **25.71** | **N/A** |
| **EGR1** | **P18146** | **-2.03** | **🡻** | **0.0001074** | **0.00439** |  | **761** | **N/A** | **0.39** | **883** | **2.38** | **N/A** |
| **SPNS2** | **Q8IVW8** | **-2.04** | **🡻** | **0.0005053** | **0.0381** |  | **761** | **N/A** | **0.53** | **883** | **2.72** | **N/A** |
| **UFC1** | **Q9Y3C8** | **-2.05** | **🡻** | **0.0003189** | **0.00834** |  | **761** | **8.54** | **N/A** | **883** | **25.93** | **N/A** |
| **DDX50** | **Q9BQ39** | **-2.07** | **🡻** | **0.0008845** | **0.0589** |  | **761** | **0.53** | **0.26** | **883** | **7.02** | **5.66** |
| **RGS2** | **P41220** | **-2.07** | **🡻** | **0.0006273** | **0.0134** |  | **761** | **12.09** | **N/A** | **883** | **2.49** | **N/A** |
| **PTK2B** | **Q14289** | **-2.08** | **🡻** | **0.0007756** | **0.0539** |  | **761** | **0.79** | **4.99** | **883** | **1.7** | **N/A** |
| **SKIV2L2** | **P42285** | **-2.10** | **🡻** | **0.0004732** | **0.0429** |  | **761** | **0.13** | **0.79** | **883** | **5.55** | **4.64** |
| **C17orf79** | **Q9NQ92** | **-2.11** | **🡻** | **0.0004805** | **0.0429** |  | **N/A** | **N/A** | **N/A** | **N/A** | **N/A** | **N/A** |
| **SPAG9** | **O60271** | **-2.11** | **🡻** | **8.80E-06** | **0.00331** |  | **761** | **6.96** | **N/A** | **883** | **16.87** | **1.13** |
| **STAB1** | **Q9NY15** | **-2.11** | **🡻** | **9.20E-06** | **0.00115** |  | **761** | **0.13** | **0.39** | **883** | **1.93** | **N/A** |
| **RAN** | **P62826** | **-2.12** | **🡻** | **4.24E-05** | **0.0115** |  | **761** | **0.13** | **0.53** | **883** | **8.38** | **N/A** |
| **PUF60** | **Q9UHX1** | **-2.14** | **🡻** | **3.83E-05** | **0.00236** |  | **761** | **9.72** | **N/A** | **883** | **20.61** | **N/A** |
| **GART** | **P22102** | **-2.15** | **🡻** | **0.0008952** | **0.0517** |  | **761** | **0.13** | **N/A** | **883** | **12** | **N/A** |
| **SNRNP200** | **O75643** | **-2.15** | **🡻** | **0.000785** | **0.0605** |  | **761** | **0.53** | **N/A** | **883** | **6.68** | **0.45** |
| **WAPAL** | **Q7Z5K2** | **-2.15** | **🡻** | **0.000665** | **0.0489** |  | **761** | **0.13** | **0.13** | **883** | **3.62** | **1.05** |
| **ARHGDIA** | **P52565** | **-2.16** | **🡻** | **0.0007601** | **0.0149** |  | **761** | **2.1** | **N/A** | **883** | **10.76** | **N/A** |
| **USP12** | **O75317** | **-2.16** | **🡻** | **0.0005279** | **0.0332** |  | **761** | **1.05** | **0.92** | **883** | **3.74** | **N/A** |
| **HLA-B** | **P01889** | **-2.17** | **🡻** | **8.11E-05** | **0.0151** |  | **761** | **0.26** | **N/A** | **883** | **5.66** | **N/A** |
| **WDR47** | **O94967** | **-2.19** | **🡻** | **0.0001228** | **0.0156** |  | **761** | **0.39** | **N/A** | **883** | **4.74** | **1.25** |
| **ABAT** | **P80404** | **-2.20** | **🡻** | **0.0005873** | **0.0537** |  | **761** | **3.81** | **N/A** | **883** | **12.8** | **N/A** |
| **SRPR** | **P08240** | **-2.20** | **🡻** | **3.07E-05** | **0.0096** |  | **761** | **0.39** | **1.05** | **883** | **4.76** | **2.49** |
| **STAT1** | **P42224** | **-2.21** | **🡻** | **1.70E-06** | **0.000754** |  | **761** | **0.66** | **N/A** | **883** | **7.25** | **N/A** |
| **STX18** | **Q9P2W9** | **-2.21** | **🡻** | **0.0003318** | **0.0328** |  | **761** | **0.26** | **0.53** | **883** | **2.27** | **4.19** |
| **CHMP1B** | **Q7LBR1** | **-2.22** | **🡻** | **0.0009128** | **0.0517** |  | **761** | **0.53** | **0.39** | **883** | **9.29** | **5.32** |
| **TSEN2** | **Q8NCE0** | **-2.23** | **🡻** | **4.20E-06** | **0.000668** |  | **761** | **0.66** | **N/A** | **883** | **9.51** | **2.72** |
| **VPS37B** | **Q9H9H4** | **-2.23** | **🡻** | **1.74E-05** | **0.00231** |  | **761** | **N/A** | **0.26** | **883** | **5.78** | **N/A** |
| **CCNDBP1** | **O95273** | **-2.23** | **🡻** | **0.0002516** | **0.0278** |  | **761** | **0.26** | **0.26** | **883** | **2.38** | **7.93** |
| **SOD1** | **P00441** | **-2.26** | **🡻** | **0.0002996** | **0.0317** |  | **761** | **0.26** | **N/A** | **883** | **6.91** | **N/A** |
| **BCL11A** | **Q9H165** | **-2.27** | **🡻** | **3.80E-06** | **0.000654** | **+** | **761** | **0.66** | **N/A** | **883** | **9.4** | **N/A** |
| **SH3BP1** | **Q9Y3L3** | **-2.29** | **🡻** | **0.000191** | **0.00614** |  | **761** | **0.26** | **0.26** | **883** | **4.3** | **N/A** |
| **FUT8** | **Q9BYC5** | **-2.29** | **🡻** | **< 1e-07** | **< 1e-07** |  | **761** | **N/A** | **0.26** | **883** | **3.74** | **N/A** |
| **TBC1D10C** | **Q8IV04** | **-2.33** | **🡻** | **0.0003061** | **0.0317** |  | **761** | **4.99** | **N/A** | **883** | **1.93** | **N/A** |
| **DSN1** | **Q9H410** | **-2.36** | **🡻** | **0.0007004** | **0.0143** |  | **761** | **1.58** | **N/A** | **883** | **15.86** | **N/A** |
| **NOC2L** | **Q9Y3T9** | **-2.36** | **🡻** | **3.00E-07** | **0.000207** |  | **761** | **N/A** | **0.26** | **883** | **6.23** | **N/A** |
| **RSC1A1** | **Q92681** | **-2.36** | **🡻** | **1.53E-05** | **0.00154** |  | **761** | **0.13** | **0.53** | **883** | **3.74** | **N/A** |
| **AMN1** | **Q8IY45** | **-2.40** | **🡻** | **6.19E-05** | **0.0129** |  | **761** | **2.89** | **N/A** | **883** | **4.3** | **N/A** |
| **TYK2** | **P29597** | **-2.41** | **🡻** | **0.0009588** | **0.0531** |  | **761** | **0.92** | **N/A** | **883** | **5.44** | **1.59** |
| **BAZ1B** | **Q9UIG0** | **-2.42** | **🡻** | **5.40E-06** | **0.00196** |  | **761** | **1.18** | **0.13** | **883** | **8.95** | **0.34** |
| **BIN1** | **O00499** | **-2.42** | **🡻** | **0.0001102** | **0.0148** |  | **761** | **0.26** | **N/A** | **883** | **1.7** | **N/A** |
| **LUC7L3** | **O95232** | **-2.43** | **🡻** | **0.0008966** | **0.0517** |  | **761** | **7.1** | **N/A** | **883** | **7.93** | **N/A** |
| **RNF146** | **Q9NTX7** | **-2.43** | **🡻** | **6.25E-05** | **0.00319** |  | **761** | **1.31** | **0.26** | **883** | **5.55** | **5.21** |
| **ACOT9** | **Q9Y305** | **-2.44** | **🡻** | **0.0009517** | **0.0664** |  | **761** | **1.05** | **1.18** | **883** | **8.38** | **N/A** |
| **OTUB1** | **Q96FW1** | **-2.44** | **🡻** | **2.86E-05** | **0.0096** |  | **761** | **0.39** | **N/A** | **883** | **5.32** | **0.23** |
| **CTSH** | **P09668** | **-2.44** | **🡻** | **0.0008808** | **0.0517** |  | **761** | **0.39** | **0.39** | **883** | **3.51** | **N/A** |
| **MTA2** | **O94776** | **-2.46** | **🡻** | **0.0007376** | **0.0473** |  | **761** | **0.79** | **N/A** | **883** | **7.59** | **0.68** |
| **CYLD** | **Q9NQC7** | **-2.46** | **🡻** | **0.0001367** | **0.0185** | **+** | **761** | **0.53** | **0.66** | **883** | **2.15** | **N/A** |
| **CHMP2B** | **Q9UQN3** | **-2.48** | **🡻** | **0.0007518** | **0.0148** |  | **761** | **0.39** | **0.13** | **883** | **8.15** | **2.72** |
| **C20orf20** | **Q9NV56** | **-2.49** | **🡻** | **0.0001898** | **0.00614** |  | **N/A** | **N/A** | **N/A** | **N/A** | **N/A** | **N/A** |
| **SEPT6** | **Q14141** | **-2.50** | **🡻** | **0.0003364** | **0.0286** |  | **761** | **0.39** | **1.18** | **883** | **4.42** | **N/A** |
| **ACIN1** | **Q9UKV3** | **-2.50** | **🡻** | **1.87E-05** | **0.0096** |  | **761** | **0.79** | **0.13** | **883** | **3.06** | **2.72** |
| **POGK** | **Q9P215** | **-2.51** | **🡻** | **0.0009279** | **0.0246** |  | **761** | **6.83** | **N/A** | **883** | **33.75** | **0.23** |
| **FGB** | **P02675** | **-2.51** | **🡻** | **0.0001982** | **0.00934** |  | **761** | **0.53** | **0.53** | **883** | **0.91** | **N/A** |
| **GATA6** | **Q92908** | **-2.53** | **🡻** | **0.0004165** | **0.0143** |  | **761** | **0.13** | **0.26** | **883** | **3.4** | **N/A** |
| **FBRS** | **Q9HAH7** | **-2.54** | **🡻** | **0.0003125** | **0.0277** |  | **761** | **3.02** | **N/A** | **883** | **11.55** | **0.57** |
| **DDX54** | **Q8TDD1** | **-2.54** | **🡻** | **0.0002327** | **0.00682** |  | **761** | **N/A** | **0.13** | **883** | **3.74** | **0.23** |
| **ZNF131** | **P52739** | **-2.57** | **🡻** | **0.000501** | **0.0166** |  | **761** | **1.45** | **N/A** | **883** | **11.44** | **0.79** |
| **FKBP3** | **Q00688** | **-2.57** | **🡻** | **8.45E-05** | **0.00385** |  | **761** | **0.26** | **0.13** | **883** | **7.59** | **0.34** |
| **CAMSAP1** | **Q5T5Y3** | **-2.58** | **🡻** | **0.0007472** | **0.0473** |  | **761** | **0.26** | **0.39** | **883** | **6.57** | **0.79** |
| **HAUS4** | **Q9H6D7** | **-2.60** | **🡻** | **0.0004729** | **0.0108** |  | **761** | **0.79** | **0.13** | **883** | **6.46** | **2.27** |
| **ARHGAP44** | **Q17R89** | **-2.62** | **🡻** | **0.0009155** | **0.0517** |  | **761** | **0.13** | **1.58** | **N/A** | **N/A** | **N/A** |
| **NUP93** | **Q8N1F7** | **-2.62** | **🡻** | **3.59E-05** | **0.00236** |  | **761** | **0.53** | **0.66** | **883** | **6** | **N/A** |
| **MAZ** | **P56270** | **-2.64** | **🡻** | **0.0004003** | **0.0139** |  | **761** | **2.63** | **N/A** | **883** | **8.49** | **N/A** |
| **SNRNP35** | **Q16560** | **-2.64** | **🡻** | **0.0007692** | **0.0605** |  | **761** | **0.26** | **0.39** | **883** | **3.85** | **N/A** |
| **PDAP1** | **Q13442** | **-2.65** | **🡻** | **0.0009879** | **0.0253** |  | **761** | **0.26** | **0.39** | **883** | **6.34** | **N/A** |
| **ZNF160** | **Q9HCG1** | **-2.65** | **🡻** | **0.0001577** | **0.00807** |  | **761** | **1.58** | **0.13** | **883** | **5.21** | **2.94** |
| **IPO13** | **O94829** | **-2.67** | **🡻** | **0.0007515** | **0.0148** |  | **997** | **0.3** | **0.1** | **1104** | **8.06** | **1.99** |
| **AARS** | **P49588** | **-2.67** | **🡻** | **8.40E-06** | **0.00264** |  | **761** | **0.26** | **0.92** | **883** | **5.1** | **N/A** |
| **PLXNA1** | **Q9UIW2** | **-2.67** | **🡻** | **2.68E-05** | **0.00535** |  | **761** | **0.13** | **0.13** | **883** | **4.19** | **N/A** |
| **HUWE1** | **Q7Z6Z7** | **-2.68** | **🡻** | **0.0009204** | **0.0517** |  | **761** | **0.13** | **0.92** | **883** | **6.68** | **N/A** |
| **CLPTM1L** | **Q96KA5** | **-2.69** | **🡻** | **0.0004937** | **0.0111** |  | **761** | **2.1** | **0.26** | **883** | **11.89** | **0.11** |
| **MCRS1** | **Q96EZ8** | **-2.75** | **🡻** | **0.0007611** | **0.0215** |  | **761** | **0.13** | **0.13** | **883** | **4.3** | **0.11** |
| **RAC2** | **P15153** | **-2.78** | **🡻** | **1.10E-06** | **0.00103** |  | **761** | **0.26** | **0.39** | **883** | **1.81** | **N/A** |
| **ZNFX1** | **Q9P2E3** | **-2.81** | **🡻** | **0.0001083** | **0.0148** |  | **761** | **3.68** | **N/A** | **883** | **10.31** | **N/A** |
| **TTYH1** | **Q9H313** | **-2.82** | **🡻** | **0.0006962** | **0.0143** |  | **761** | **1.71** | **0.13** | **883** | **5.44** | **N/A** |
| **MGEA5** | **O60502** | **-2.82** | **🡻** | **0.000447** | **0.0343** |  | **761** | **0.13** | **0.39** | **883** | **3.62** | **2.15** |
| **LAP3** | **P28838** | **-2.83** | **🡻** | **0.0009146** | **0.0245** |  | **761** | **0.39** | **0.66** | **883** | **4.87** | **N/A** |
| **MRPL27** | **Q8IXM3** | **-2.84** | **🡻** | **0.0002916** | **0.0114** |  | **761** | **7.1** | **N/A** | **883** | **11.1** | **N/A** |
| **PGAM1** | **P18669** | **-2.87** | **🡻** | **0.0007001** | **0.0143** |  | **761** | **0.26** | **N/A** | **883** | **7.02** | **N/A** |
| **EEF1A2** | **Q05639** | **-2.87** | **🡻** | **8.55E-05** | **0.00576** |  | **761** | **3.42** | **N/A** | **883** | **6.91** | **N/A** |
| **RRBP1** | **Q9P2E9** | **-2.89** | **🡻** | **5.00E-07** | **0.000625** |  | **761** | **1.18** | **0.26** | **883** | **7.93** | **N/A** |
| **HSPA9** | **P38646** | **-2.93** | **🡻** | **3.17E-05** | **0.0023** |  | **761** | **N/A** | **0.39** | **883** | **9.97** | **0.45** |
| **ATP5SL** | **Q9NW81** | **-2.95** | **🡻** | **4.76E-05** | **0.00376** |  | **761** | **0.66** | **N/A** | **883** | **5.89** | **4.08** |
| **RAF1** | **P04049** | **-2.96** | **🡻** | **0.0007202** | **0.0209** | **+** | **761** | **0.66** | **N/A** | **883** | **11.78** | **2.38** |
| **ACAT2** | **O75908** | **-2.98** | **🡻** | **2.00E-07** | **0.000165** |  | **761** | **0.92** | **N/A** | **883** | **6.46** | **N/A** |
| **ZC3H13** | **Q5T200** | **-3.03** | **🡻** | **1.10E-06** | **0.000284** |  | **761** | **0.13** | **N/A** | **883** | **4.3** | **1.7** |
| **PSME1** | **Q06323** | **-3.04** | **🡻** | **0.000183** | **0.00904** |  | **761** | **1.05** | **0.13** | **883** | **4.98** | **0.11** |
| **KIF1C** | **O43896** | **-3.04** | **🡻** | **0.0001401** | **0.00776** |  | **761** | **0.13** | **0.66** | **883** | **3.96** | **3.85** |
| **RABEPK** | **Q7Z6M1** | **-3.06** | **🡻** | **0.000203** | **0.0213** |  | **761** | **0.92** | **0.13** | **883** | **4.76** | **1.7** |
| **BSDC1** | **Q9NW68** | **-3.13** | **🡻** | **9.98E-05** | **0.00614** |  | **761** | **0.39** | **0.13** | **883** | **4.87** | **3.85** |
| **CPNE6** | **O95741** | **-3.17** | **🡻** | **1.00E-06** | **0.000276** |  | **761** | **1.05** | **0.13** | **883** | **2.38** | **N/A** |
| **PJA2** | **O43164** | **-3.20** | **🡻** | **8.00E-07** | **0.000532** |  | **761** | **0.13** | **0.53** | **883** | **3.4** | **2.94** |
| **GGA3** | **Q9NZ52** | **-3.25** | **🡻** | **2.11E-05** | **0.00519** |  | **761** | **3.94** | **N/A** | **883** | **15.86** | **3.06** |
| **HLA-C** | **P04222** | **-3.25** | **🡻** | **1.95E-05** | **0.00166** |  | **761** | **0.26** | **N/A** | **883** | **4.53** | **N/A** |
| **GNAI2** | **P04899** | **-3.26** | **🡻** | **0.0001524** | **0.0164** |  | **761** | **N/A** | **0.26** | **883** | **1.7** | **1.59** |
| **GLUL** | **P15104** | **-3.28** | **🡻** | **0.0008198** | **0.0503** |  | **761** | **8.28** | **N/A** | **883** | **15.52** | **N/A** |
| **IGF2R** | **P11717** | **-3.28** | **🡻** | **1.20E-06** | **0.000599** |  | **761** | **0.13** | **0.92** | **883** | **4.19** | **0.11** |
| **PTPRS** | **Q13332** | **-3.29** | **🡻** | **0.0009433** | **0.0247** |  | **761** | **0.39** | **N/A** | **883** | **4.98** | **N/A** |
| **NEFM** | **P07197** | **-3.29** | **🡻** | **5.00E-07** | **0.000223** |  | **761** | **0.26** | **4.99** | **883** | **1.02** | **N/A** |
| **PTPRA** | **P18433** | **-3.30** | **🡻** | **1.60E-06** | **0.000348** |  | **761** | **0.66** | **0.13** | **883** | **13.36** | **7.13** |
| **NRXN2** | **P58401** | **-3.33** | **🡻** | **5.00E-06** | **0.00331** |  | **761** | **0.39** | **0.13** | **883** | **2.38** | **N/A** |
| **DHX16** | **O60231** | **-3.34** | **🡻** | **0.000142** | **0.00776** |  | **761** | **0.26** | **N/A** | **883** | **11.44** | **2.38** |
| **POLR3E** | **Q9NVU0** | **-3.36** | **🡻** | **7.38E-05** | **0.00517** |  | **761** | **3.68** | **N/A** | **883** | **17.21** | **1.47** |
| **DCTPP1** | **Q9H773** | **-3.37** | **🡻** | **0.0002252** | **0.00999** |  | **761** | **2.89** | **N/A** | **883** | **10.08** | **N/A** |
| **RIOK3** | **O14730** | **-3.38** | **🡻** | **0.0002434** | **0.0104** |  | **761** | **0.39** | **0.92** | **883** | **8.04** | **1.13** |
| **TPR** | **P12270** | **-3.42** | **🡻** | **1.16E-05** | **0.00331** | **+** | **761** | **10.78** | **N/A** | **883** | **21.29** | **0.34** |
| **ARF4** | **P18085** | **-3.45** | **🡻** | **0.0001569** | **0.0054** |  | **761** | **N/A** | **0.53** | **883** | **4.42** | **0.45** |
| **NISCH** | **Q9Y2I1** | **-3.46** | **🡻** | **5.45E-05** | **0.0087** |  | **761** | **0.13** | **0.39** | **883** | **1.25** | **3.62** |
| **MYH14** | **Q7Z406** | **-3.47** | **🡻** | **9.00E-06** | **0.00115** |  | **761** | **1.31** | **N/A** | **883** | **6.23** | **N/A** |
| **UBA52** | **P62987** | **-3.49** | **🡻** | **0.0003637** | **0.0294** |  | **761** | **1.05** | **N/A** | **883** | **3.4** | **N/A** |
| **PLCE1** | **Q9P212** | **-3.50** | **🡻** | **4.00E-07** | **7.79E-05** |  | **761** | **0.26** | **0.39** | **883** | **3.17** | **N/A** |
| **PKD1** | **P98161** | **-3.50** | **🡻** | **0.000147** | **0.00781** |  | **761** | **5.26** | **N/A** | **883** | **3.06** | **N/A** |
| **MAP7D3** | **Q8IWC1** | **-3.54** | **🡻** | **0.0008301** | **0.0226** |  | **761** | **0.26** | **1.05** | **883** | **8.38** | **N/A** |
| **FZR1** | **Q9UM11** | **-3.55** | **🡻** | **2.95E-05** | **0.00254** |  | **761** | **0.13** | **0.13** | **883** | **3.4** | **1.02** |
| **TBL1X** | **O60907** | **-3.56** | **🡻** | **2.14E-05** | **0.00177** |  | **761** | **0.39** | **1.45** | **883** | **7.13** | **N/A** |
| **HOXB3** | **P14651** | **-3.59** | **🡻** | **0.0002757** | **0.011** |  | **761** | **3.81** | **0.26** | **883** | **2.83** | **N/A** |
| **SEC13** | **P55735** | **-3.61** | **🡻** | **7.41E-05** | **0.0107** |  | **761** | **1.31** | **N/A** | **883** | **10.42** | **0.11** |
| **MAP3K3** | **Q99759** | **-3.61** | **🡻** | **0.000354** | **0.0294** |  | **761** | **7.62** | **0.26** | **883** | **6.68** | **0.34** |
| **SLC25A6** | **P12236** | **-3.65** | **🡻** | **0.0001457** | **0.0162** |  | **761** | **0.26** | **1.05** | **883** | **3.74** | **N/A** |
| **PSMD13** | **Q9UNM6** | **-3.73** | **🡻** | **8.94E-05** | **0.0118** |  | **761** | **0.13** | **0.53** | **883** | **3.28** | **N/A** |
| **RNF213** | **Q63HN8** | **-3.75** | **🡻** | **0.0003689** | **0.0294** | **+** | **761** | **2.37** | **N/A** | **883** | **10.08** | **N/A** |
| **CCDC106** | **Q9BWC9** | **-3.76** | **🡻** | **0.0001142** | **0.0069** |  | **997** | **2.11** | **0.2** | **1104** | **4.44** | **N/A** |
| **DDX39A** | **O00148** | **-3.77** | **🡻** | **0.0001341** | **0.0157** |  | **761** | **0.79** | **N/A** | **N/A** | **N/A** | **N/A** |
| **KANSL2** | **Q9H9L4** | **-3.81** | **🡻** | **< 1e-07** | **< 1e-07** |  | **761** | **0.13** | **N/A** | **N/A** | **N/A** | **N/A** |
| **NAGLU** | **P54802** | **-3.87** | **🡻** | **9.90E-06** | **0.00119** |  | **761** | **0.66** | **0.13** | **N/A** | **N/A** | **N/A** |
| **MUC2** | **Q02817** | **-3.87** | **🡻** | **6.15E-05** | **0.00944** |  |  | **N/A** | **N/A** | **N/A** | **N/A** | **N/A** |
| **MINK1** | **Q8N4C8** | **-3.89** | **🡻** | **4.41E-05** | **0.00742** |  | **1143** | **0.17** | **N/A** | **N/A** | **N/A** | **N/A** |
| **NLE1** | **Q9NVX2** | **-3.96** | **🡻** | **1.20E-06** | **0.000599** |  | **761** | **1.18** | **0.39** | **883** | **6.91** | **0.45** |
| **PNPT1** | **Q8TCS8** | **-4.04** | **🡻** | **4.80E-06** | **0.000835** |  | **761** | **0.53** | **N/A** | **883** | **11.66** | **N/A** |
| **UBR4** | **Q5T4S7** | **-4.04** | **🡻** | **4.35E-05** | **0.00742** |  | **761** | **N/A** | **0.53** | **883** | **2.38** | **1.25** |
| **ACTN4** | **O43707** | **-4.06** | **🡻** | **2.37E-05** | **0.00519** |  | **761** | **1.71** | **N/A** | **883** | **8.49** | **0.11** |
| **TRMT2A** | **Q8IZ69** | **-4.23** | **🡻** | **0.0001513** | **0.00538** |  | **761** | **0.13** | **0.13** | **883** | **3.85** | **N/A** |
| **SEPT1** | **Q8WYJ6** | **-4.28** | **🡻** | **< 1e-07** | **< 1e-07** |  | **761** | **2.89** | **N/A** | **883** | **1.59** | **N/A** |
| **STRAP** | **Q9Y3F4** | **-4.33** | **🡻** | **0.0005362** | **0.0119** |  | **761** | **1.84** | **0.13** | **883** | **10.31** | **0.34** |
| **NUMA1** | **Q14980** | **-4.33** | **🡻** | **2.00E-07** | **6.37E-05** | **+** | **761** | **2.5** | **N/A** | **883** | **5.78** | **1.36** |
| **AP2A1** | **O95782** | **-4.33** | **🡻** | **7.60E-06** | **0.00102** |  | **761** | **1.58** | **0.13** | **883** | **5.55** | **0.68** |
| **GMPPB** | **Q9Y5P6** | **-4.38** | **🡻** | **4.46E-05** | **0.00742** |  | **761** | **N/A** | **0.26** | **883** | **3.62** | **N/A** |
| **STRN4** | **Q9NRL3** | **-4.41** | **🡻** | **2.00E-07** | **0.000165** |  | **761** | **0.39** | **0.13** | **883** | **4.53** | **0.45** |
| **ANTXR1** | **Q9H6X2** | **-4.47** | **🡻** | **7.65E-05** | **0.0107** |  | **761** | **0.26** | **N/A** | **883** | **5.21** | **N/A** |
| **THAP7** | **Q9BT49** | **-4.50** | **🡻** | **3.67E-05** | **0.00697** |  | **997** | **0.2** | **0.1** | **1104** | **3.44** | **N/A** |
| **AMBRA1** | **Q9C0C7** | **-4.52** | **🡻** | **1.00E-07** | **9.98E-05** |  | **761** | **0.53** | **0.13** | **883** | **5.32** | **4.98** |
| **GBAS** | **O75323** | **-4.55** | **🡻** | **7.20E-06** | **0.00101** |  | **761** | **1.31** | **0.13** | **883** | **6.57** | **0.11** |
| **AKT1** | **P31749** | **-4.61** | **🡻** | **5.30E-06** | **0.000838** | **+** | **761** | **0.53** | **0.26** | **883** | **7.93** | **0.91** |
| **VIM** | **P08670** | **-4.62** | **🡻** | **1.00E-07** | **3.51E-05** |  | **761** | **1.58** | **N/A** | **883** | **2.94** | **N/A** |
| **DFFA** | **O00273** | **-4.70** | **🡻** | **0.0004036** | **0.0316** |  | **761** | **0.26** | **0.39** | **883** | **4.64** | **0.11** |
| **BEX4** | **Q9NWD9** | **-4.84** | **🡻** | **1.60E-06** | **0.000348** |  | **761** | **N/A** | **1.71** | **883** | **7.02** | **N/A** |
| **KDM5A** | **P29375** | **-5.12** | **🡻** | **0.000194** | **0.00614** | **+** | **761** | **3.02** | **N/A** | **883** | **7.93** | **0.79** |
| **FBXL5** | **Q9UKA1** | **-5.42** | **🡻** | **0.0006027** | **0.043** |  | **761** | **0.26** | **0.66** | **883** | **2.94** | **6** |
| **MGA** | **O43451** | **-5.44** | **🡻** | **< 1e-07** | **< 1e-07** |  | **761** | **0.26** | **0.26** | **883** | **2.83** | **1.59** |
| **CINP** | **Q9BW66** | **-5.55** | **🡻** | **< 1e-07** | **< 1e-07** |  | **761** | **0.79** | **0.26** | **883** | **5.21** | **0.34** |
| **EIF3G** | **O75821** | **-6.30** | **🡻** | **1.05E-05** | **0.00119** |  | **761** | **0.79** | **N/A** | **883** | **3.62** | **N/A** |
| **HNRNPA1** | **P09651** | **-6.52** | **🡻** | **< 1e-07** | **< 1e-07** |  | **761** | **N/A** | **0.13** | **883** | **4.19** | **N/A** |
| **RPLP0P2** | **#N/A** | **-6.86** | **🡻** | **4.20E-06** | **0.00168** |  | **761** | **N/A** | **0.26** | **883** | **4.53** | **N/A** |
| **TAOK1** | **Q7L7X3** | **-7.33** | **🡻** | **< 1e-07** | **< 1e-07** |  | **1143** | **2.36** | **0.09** | **N/A** | **N/A** | **N/A** |
| **MCM3AP** | **O60318** | **-7.57** | **🡻** | **8.60E-06** | **0.00264** |  | **761** | **0.66** | **N/A** | **883** | **9.97** | **0.45** |
| **MED23** | **Q9ULK4** | **-7.88** | **🡻** | **< 1e-07** | **< 1e-07** |  | **761** | **1.45** | **0.39** | **883** | **3.17** | **0.68** |
| **TRIP12** | **Q14669** | **-8.21** | **🡻** | **< 1e-07** | **< 1e-07** |  | **761** | **0.13** | **0.26** | **883** | **2.94** | **0.45** |
| **CDT1** | **Q9H211** | **-9.99** | **🡻** | **< 1e-07** | **< 1e-07** |  | **761** | **N/A** | **1.05** | **883** | **3.4** | **N/A** |

Complete list of differentially reactive proteins (breast cancer vs. healthy controls). Fold changes, false discovery rates (FDR) and p-values are shown. COSMIC database gene census concordances are indicated with plus signs. Copy number variation and gene expression data show the percentage of samples (in the given number of samples in COSMIC) that were over-/under-expressed; or the percentage of samples where CNV gain or losses were observed.
