# Supplementary material for: IgG based immunome analyses of breast cancer patients reveal underlying signaling pathways
Source: Oncotarget. 2019 May 28;10(37):3491–505. doi: 10.18632/oncotarget.26834 (PMC6544406; doi:10.18632/oncotarget.26834)
Supplement: Supplementary file 3 [file oncotarget-10-3491-s003.docx]

**Supplementary Table 2: Complete list of genes associated with signaling pathways and terms mentioned in the article**

**Supplementary Table 2A: Differentially reactive antigens, which associated with ingenuity canonical pathways**

| **Ingenuity Canonical Pathways** | **p-value** | **Ratio** |  |
| --- | --- | --- | --- |
| FcÎ³ Receptor-mediated Phagocytosis in Macrophages and Monocytes | 4.17E-05 | 1.67E-01 | NCK2,RAC2,PXN,YES1,ACTR3,AKT1,PTK2B,EZR,RPS6KB2,PIK3R2,VASP,PRKCZ |
| CTLA4 Signaling in Cytotoxic T Lymphocytes | 5.75E-04 | 1.58E-01 | AP2A1,AKT1,HLA-A,HLA-C,PPP2R5D,CLTC,HLA-B,PIK3CD,PIK3R2 |
| EIF2 Signaling | 6.61E-04 | 1.05E-01 | RPL24,PABPC1,RAF1,EIF2B4,RPL17,EIF4A2,RPS17/RPS17L,RPL7,EIF3G,AKT1,EIF3D,UBA52,EIF3A,EIF2S3,PIK3CD,PIK3R2 |
| VEGF Signaling | 1.02E-03 | 1.35E-01 | RAF1,PXN,EIF2B4,AKT1,PTK2B,EIF2S3,PIK3CD,PIK3R2,ACTN4,ARNT |
| Regulation of eIF4 and p70S6K Signaling | 1.05E-03 | 1.09E-01 | PABPC1,RAF1,EIF2B4,PPP2R5D,EIF4A2,RPS17/RPS17L,PRKCZ,EIF3G,AKT1,EIF3D,EIF2S3,EIF3A,PIK3CD,PIK3R2 |
| CNTF Signaling | 1.26E-03 | 1.75E-01 | RAF1,AKT1,TYK2,RPS6KB2,PIK3CD,PIK3R2,STAT1 |
| IL-15 Production | 1.51E-03 | 2.38E-01 | RELA,PTK2B,TYK2,STAT1,PRKCZ |
| SAPK/JNK Signaling | 2.82E-03 | 1.27E-01 | MINK1,RAC2,TRADD,DAXX,PIK3CD,PIK3R2,MAP3K4,MAPK8IP3,MAP3K3 |
| Role of JAK1, JAK2 and TYK2 in Interferon Signaling | 2.95E-03 | 2.67E-01 | RAF1,RELA,TYK2,STAT1 |
| Virus Entry via Endocytic Pathways | 3.47E-03 | 1.23E-01 | RAC2,AP2A1,HLA-A,HLA-C,CLTC,HLA-B,PIK3CD,PIK3R2,PRKCZ |
| Leukocyte Extravasation Signaling | 3.98E-03 | 9.77E-02 | RAC2,PXN,PTK2B,MMP14,MAP3K4,PRKCZ,GNAI2,EZR,PIK3CD,PIK3R2,ACTN4,ARHGAP1,VASP |
| IL-9 Signaling | 5.75E-03 | 1.79E-01 | RELA,BCL3,PIK3CD,PIK3R2,STAT1 |
| PI3K/AKT Signaling | 5.75E-03 | 1.02E-01 | RAF1,RELA,AKT1,HSP90AB1,PPP2R5D,TSC2,TYK2,RPS6KB2,PIK3CD,PIK3R2,PRKCZ |
| 14-3-3-mediated Signaling | 6.17E-03 | 1.06E-01 | RAF1,AKT1,PLCE1,TSC2,VIM,PIK3CD,PIK3R2,PDCD6IP,TUBA1B,PRKCZ |
| PTEN Signaling | 6.61E-03 | 1.05E-01 | RAF1,RAC2,RELA,AKT1,RPS6KB2,PIK3CD,PIK3R2,MCRS1,IGF2R,PRKCZ |
| Ceramide Signaling | 6.92E-03 | 1.19E-01 | CTSD,RAF1,RELA,AKT1,PPP2R5D,PIK3CD,PIK3R2,PRKCZ |
| Reelin Signaling in Neurons | 7.24E-03 | 1.30E-01 | YES1,AKT1,NDEL1,PIK3CD,PIK3R2,MAPK8IP3,PAFAH1B3 |
| ILK Signaling | 7.41E-03 | 9.09E-02 | RELA,PXN,FN1,SNAI2,PPP2R5D,MYH14,VIM,NCK2,TGFB1I1,AKT1,PIK3CD,PIK3R2,ACTN4 |
| Protein Ubiquitination Pathway | 8.71E-03 | 7.98E-02 | FZR1,USP12,HLA-A,PSMD13,HSPA9,HLA-B,PSMD6,DNAJC10,PSME1,UBE2L3,HSP90AB1,UBE2B,HLA-C,PSMA2,NEDD4L,PSMC3,HSPB1 |
| RANK Signaling in Osteoclasts | 8.91E-03 | 1.14E-01 | RAF1,RELA,AKT1,PTK2B,PIK3CD,PIK3R2,MAP3K4,MAP3K3 |
| Systemic Lupus Erythematosus Signaling | 9.12E-03 | 9.57E-02 | SNRNP35,AKT1,HLA-A,HLA-C,PRPF8,HLA-B,SNRNP200,SF3B4,PIK3CD,PIK3R2,PRPF31 |
| Role of Tissue Factor in Cancer | 1.00E-02 | 1.05E-01 | P4HB,YES1,AKT1,PTK2B,EGR1,FGB,PIK3CD,PIK3R2,CYR61 |
| Dendritic Cell Maturation | 1.07E-02 | 9.80E-02 | COL1A1,RELA,AKT1,PLCE1,HLA-A,HLA-C,HLA-B,PIK3CD,PIK3R2,STAT1 |
| NGF Signaling | 1.35E-02 | 1.00E-01 | RAF1,RELA,AKT1,RPS6KB2,PIK3CD,PIK3R2,MAP3K4,MAP3K3,PRKCZ |
| IL-15 Signaling | 1.82E-02 | 1.20E-01 | RAF1,RELA,AKT1,TYK2,PIK3CD,PIK3R2 |
| Cytotoxic T Lymphocyte-mediated Apoptosis of Target Cells | 1.95E-02 | 1.60E-01 | HLA-A,HLA-C,HLA-B,DFFA |
| mTOR Signaling | 2.14E-02 | 7.93E-02 | PPP2R5D,EIF4A2,RPS17/RPS17L,PRKCZ,ATG13,EIF3G,AKT1,EIF3D,TSC2,RPS6KB2,EIF3A,PIK3CD,PIK3R2 |
| Role of PI3K/AKT Signaling in the Pathogenesis of Influenza | 2.57E-02 | 1.25E-01 | GNAI2,RELA,AKT1,PIK3CD,PIK3R2 |
| HGF Signaling | 2.69E-02 | 9.41E-02 | RAF1,PXN,AKT1,PIK3CD,PIK3R2,MAP3K4,MAP3K3,PRKCZ |
| Caveolar-mediated Endocytosis Signaling | 2.75E-02 | 1.09E-01 | COPZ1,ARCN1,RAB5C,HLA-A,HLA-C,HLA-B |
| Integrin Signaling | 2.95E-02 | 7.79E-02 | NCK2,RAF1,RAC2,PXN,ACTR3,AKT1,ARF4,PIK3CD,PIK3R2,ACTN4,NEDD9,VASP |
| Rac Signaling | 3.02E-02 | 9.20E-02 | RAF1,RELA,ACTR3,PTK2B,PIK3CD,PIK3R2,PARD3,PRKCZ |
| IGF-1 Signaling | 3.16E-02 | 9.09E-02 | RAF1,PXN,AKT1,RPS6KB2,PIK3CD,PIK3R2,CYR61,PRKCZ |
| Autoimmune Thyroid Disease Signaling | 3.24E-02 | 1.76E-01 | HLA-A,HLA-C,HLA-B |
| JAK/Stat Signaling | 3.47E-02 | 1.03E-01 | RAF1,AKT1,TYK2,PIK3CD,PIK3R2,STAT1 |
| P2Y Purigenic Receptor Signaling Pathway | 3.55E-02 | 8.89E-02 | GNAI2,RAF1,RELA,AKT1,PLCE1,PIK3CD,PIK3R2,PRKCZ |
| OX40 Signaling Pathway | 3.63E-02 | 1.33E-01 | RELA,HLA-A,HLA-C,HLA-B |
| B Cell Receptor Signaling | 3.63E-02 | 8.06E-02 | RAF1,RAC2,RELA,AKT1,EGR1,RPS6KB2,PIK3CD,PIK3R2,MAP3K4,MAP3K3 |
| IL-2 Signaling | 3.72E-02 | 1.14E-01 | RAF1,AKT1,PTK2B,PIK3CD,PIK3R2 |
| Erythropoietin Signaling | 3.72E-02 | 1.02E-01 | RAF1,RELA,AKT1,PIK3CD,PIK3R2,PRKCZ |
| Hypoxia Signaling in the Cardiovascular System | 3.72E-02 | 1.02E-01 | P4HB,UBE2L3,AKT1,UBE2B,HSP90AB1,ARNT |
| Type I Diabetes Mellitus Signaling | 3.80E-02 | 9.33E-02 | TRADD,RELA,HLA-A,HLA-C,HLA-B,STAT1,CPE |
| NF-ÎºB Activation by Viruses | 3.98E-02 | 1.00E-01 | RAF1,RELA,AKT1,PIK3CD,PIK3R2,PRKCZ |
| PI3K Signaling in B Lymphocytes | 4.27E-02 | 8.60E-02 | CD81,RAF1,RELA,AKT1,PLCE1,PIK3CD,PIK3R2,PRKCZ |
| IL-6 Signaling | 4.68E-02 | 8.42E-02 | COL1A1,RAF1,RELA,AKT1,PIK3CD,PIK3R2,MAPKAPK2,HSPB1 |
| Acute Myeloid Leukemia Signaling | 4.90E-02 | 9.52E-02 | RAF1,RELA,AKT1,RPS6KB2,PIK3CD,PIK3R2 |
| p70S6K Signaling | 4.90E-02 | 8.33E-02 | GNAI2,RAF1,AKT1,PLCE1,PPP2R5D,PIK3CD,PIK3R2,PRKCZ |

**Supplementary Table 2B: Differentially reactive molecules associated with PathwayCommons terms**

| **PathwayName** | **#Proteins** | **p-value** | **Ratio of enrichment** | Molecules |
| --- | --- | --- | --- | --- |
| KitReceptor | 11 | 9.68E-06 | 4.49 | CISH,PLCG1,AKT1,PTPRU,RAF1,KHDRBS1,CLTC,PIK3R2,STAT1,PLCE1,YES1 |
| Adaptive Immune System | 25 | 0.0001 | 2.21 | SEC13,UBA52,AKT1,LCK,PSMA2,RAF1,PTPRA,PSMC3,PSME1,LCP2,HLA-A,  PSMC4,HLA-B,PLCG1,VASP,PSMD13,SPTBN1,FZR1,PIK3R2,PSME4,HLA-C,PSMD6,CD81,RELA,SPTAN1 |
| Signaling by Aurora kinases | 14 | 8.56E-05 | 3.1 | NDEL1,CYLD,AKT1,LCK,FZR1,NDC80,NPM1,VIM,RAN,CDC25B,BCL3,RELA,PPP2R5D,AURKAIP1 |
| IL2-mediated signaling events | 15 | 0.0002 | 2.74 | MAPKAPK2,CISH,CYLD,AKT1,LCK,RAF1,PRKCZ,STAT1,PTK2B,BCL3,RAN,DOK2,RELA,PPP2R5D,EIF3A |
| Aurora A signaling | 11 | 0.0002 | 3.43 | NDEL1,CYLD,AKT1,LCK,FZR1,RAN,CDC25B,BCL3,RELA,PPP2R5D,AURKAIP1 |
| Beta1 integrin cell surface interactions | 77 | 0.0018 | 1.37 | CYLD,TSC2,RAF1,KDR,PTPRA,ACTR3,NPM1,FOSL2,E4F1,RAB11FIP3,VTN,  ZFYVE28,LMO4,SPTBN1,MMP14,BCL3,CDC25B,RAN,RELA,CPE,STRAP,SNAI2,  DOCK4,MAP3K3,SLC3A2,CP,AKT1,BGLAP,TAOK1,TYK2,ARF4,MAP3K4,PLCG1,  DAXX,CLTC,PRKCZ,NCK2,ARHGDIA,STAT1,PTPN7,ACTN4,PTK2B,HSPB1,ARNT,  TGFB1I1,HNRNPA1,BIN1,EGR1,MAPK8IP3,NEDD4L,GGA3,COL6A3,HLA-A,  LAMB3,CISH,VASP,HUWE1,CYR61,CTSD,GNAI2,PXN,ARAP2,LCK,FHL2,RUNX3,  YES1,PKM,TAF4B,PRDX1,MAPKAPK2,SPAG9,PGK1,AP2A1,FGB,TBL1X,CD81,DOK2 |
| Arf6 downstream pathway | 73 | 0.0027 | 1.36 | CYLD,TSC2,RAF1,KDR,PTPRA,ACTR3,NPM1,FOSL2,E4F1,RAB11FIP3,VTN,ZFYVE28,LMO4,SPTBN1,MMP14,  BCL3,CDC25B,RAN,RELA,CPE,STRAP,SNAI2,DOCK4,MAP3K3,SLC3A2,CP,AKT1,BGLAP,TAOK1,TYK2,ARF4,  MAP3K4,PLCG1,DAXX,CLTC,PRKCZ,NCK2,ARHGDIA,STAT1,PTPN7,ACTN4,PTK2B,HSPB1,ARNT,TGFB1I1,  HNRNPA1,BIN1,EGR1,MAPK8IP3,NEDD4L,GGA3,HLA-A,VASP,CYR61,HUWE1,CTSD,GNAI2,PXN,ARAP2,LCK,  FHL2,RUNX3,YES1,PKM,TAF4B,PRDX1,MAPKAPK2,SPAG9,PGK1,AP2A1,FGB,TBL1X,DOK2 |
| PDGF receptor signaling network  LKB1 signaling events | 73 | 0.0027 | 1.36 | CYLD,TSC2,RAF1,KDR,PTPRA,ACTR3,NPM1,FOSL2,E4F1,RAB11FIP3,VTN,ZFYVE28,LMO4,SPTBN1,MMP14,  BCL3,CDC25B,RAN,RELA,CPE,STRAP,SNAI2,DOCK4,MAP3K3,SLC3A2,CP,AKT1,BGLAP,TAOK1,TYK2,ARF4,  MAP3K4,PLCG1,DAXX,CLTC,PRKCZ,NCK2,ARHGDIA,STAT1,PTPN7,ACTN4,PTK2B,HSPB1,ARNT,TGFB1I1,  HNRNPA1,BIN1,EGR1,MAPK8IP3,NEDD4L,GGA3,HLA-A,VASP,CYR61,HUWE1,CTSD,GNAI2,PXN,ARAP2,LCK,  FHL2,RUNX3,YES1,PKM,TAF4B,PRDX1,MAPKAPK2,SPAG9,PGK1,AP2A1,FGB,TBL1X,DOK2 |
| LKB1 signaling events | 75 | 0.0021 | 1.37 | CYLD,TSC2,RAF1,KDR,PTPRA,ACTR3,NPM1,FOSL2,E4F1,RAB11FIP3,VTN,ZFYVE28,BRSK1,LMO4,SPTBN1,MMP14,  BCL3,CDC25B,RAN,RELA,CPE,STRAP,SNAI2,DOCK4,MAP3K3,SLC3A2,CP,AKT1,BGLAP,TAOK1,EZR,TYK2,ARF4,MAP3K4,  PLCG1,DAXX,CLTC,PRKCZ,NCK2,ARHGDIA,STAT1,PTPN7,ACTN4,PTK2B,HSPB1,ARNT,TGFB1I1,HNRNPA1,BIN1,EGR1,  MAPK8IP3,NEDD4L,GGA3,HLA-A,VASP,CYR61,HUWE1,CTSD,GNAI2,PXN,ARAP2,LCK,FHL2,RUNX3,  YES1,PKM,TAF4B,PRDX1,MAPKAPK2,SPAG9,PGK1,AP2A1,FGB,TBL1X,DOK2 |
| Destabilization of mRNA by AUF1 (hnRNP D0) | 11 | 0.0024 | 2.59 | UBA52,PSMD13,PSMA2,PSMC3,FZR1,PSME4,PSME1,PSMD6,HSPB1,PABPC1,PSMC4 |

**Supplementary Table 2C: Differentially reactive molecules associated with KEGG terms**

| GO_id | Term | Number of Genes | p-value | p-value fdr | p-value  bonferroni | |
| --- | --- | --- | --- | --- | --- | --- |
| hsa03013 | RNA transport | 17 | 2.97E-08 | 7.05E-06 | 7.05E-06 | EIF2B4,SUMO1,ACIN1,EIF3D,UPF1,SEC13,DDX20,TPR,NUP107,RAN,  EIF4A2,STRAP,PABPC1,NUP93,EIF3G,EIF3A,EEF1A2 |
| hsa04370 | VEGF signaling pathway | 10 | 5.54E-06 | 6.57E-04 | 1.31E-03 | RAC2,AKT1,RAF1,MAPKAPK2,PXN,PIK3CD,PLCG1,KDR,PIK3R2,HSPB1 |
| hsa03050 | Proteasome | 7 | 4.21E-05 | 3.33E-03 | 9.99E-03 | PSMD6,PSMC3,PSMA2,PSME4,PSMD13,PSME1,PSMC4 |
| hsa04380 | Osteoclast differentiation | 11 | 8.50E-05 | 5.04E-03 | 2.02E-02 | LCK,AKT1,TYK2,RELA,STAT1,LCP2,FHL2,CYLD,PIK3CD,PIK3R2,FOSL2 |
| hsa05146 | Amoebiasis | 10 | 1.11E-04 | 5.26E-03 | 2.63E-02 | COL1A1,FN1,RELA,MUC2,ACTN4,LAMB3,HSPB1,PIK3CD,PIK3R2,RAB5C |
| hsa04510 | Focal adhesion | 14 | 1.36E-04 | 5.38E-03 | 3.23E-02 | VTN,AKT1,COL1A1,RAF1,ACTN4,LAMB3,RAC2,PXN,COL6A3,FN1,KDR,VASP,PIK3CD,PIK3R2 |
| hsa04670 | Leukocyte transendothelial migration | 10 | 1.91E-04 | 6.46E-03 | 4.53E-02 | PLCG1,ACTN4,EZR,RAC2,PTK2B,VASP,PXN,PIK3CD,GNAI2,PIK3R2 |
| hsa04144 | Endocytosis | 13 | 4.13E-04 | 1.13E-02 | 9.79E-02 | RAB11FIP3,HLA-C,AP2A1,PARD3,ARAP2,AGAP2,CHMP2B,KDR,EHD4,VPS37B,NEDD4L,CLTC,RAB5C |
| hsa03040 | Spliceosome | 10 | 4.68E-04 | 1.13E-02 | 1.11E-01 | PRPF8,SNRNP200,PRPF31,DHX16,ACIN1,PUF60,HNRNPM,RBM25,U2AF1,SF3B4 |
| hsa04914 | Progesterone-mediated oocyte maturation | 8 | 4.84E-04 | 1.13E-02 | 1.15E-01 | AKT1,RAF1,HSP90AB1,CDC25B,PIK3CD,GNAI2,PIK3R2,FZR1 |
| hsa05212 | Pancreatic cancer | 7 | 5.43E-04 | 1.13E-02 | 1.29E-01 | RAC2,AKT1,RAF1,STAT1,PIK3CD,PIK3R2,RELA |
| hsa04660 | T cell receptor signaling pathway | 9 | 5.72E-04 | 1.13E-02 | 1.36E-01 | LCK,AKT1,RAF1,PLCG1,RELA,LCP2,NCK2,PIK3CD,PIK3R2 |
| hsa04062 | Chemokine signaling pathway | 12 | 8.22E-04 | 1.43E-02 | 1.95E-01 | AKT1,RAF1,RELA,PARD3,RASGRP2,RAC2,PTK2B,STAT1,PXN,PIK3CD,GNAI2,PIK3R2 |
| hsa04010 | MAPK signaling pathway | 15 | 8.84E-04 | 1.43E-02 | 2.10E-01 | AKT1,RAF1,RELA,MAP3K3,RASGRP2,RAC2,TAOK1,MAPK8IP2,MAPK8IP3,MAP3K4,  HSPB1,MAPKAPK2,CACNB3,PTPN7,CDC25B |
| hsa04666 | Fc gamma R-mediated phagocytosis | 8 | 9.03E-04 | 1.43E-02 | 2.14E-01 | RAC2,AKT1,RAF1,RPS6KB2,VASP,PIK3CD,PIK3R2,PLCG1, |
| hsa04662 | B cell receptor signaling pathway | 7 | 1.11E-03 | 1.64E-02 | 2.62E-01 | RAC2,CD81,AKT1,RAF1,PIK3CD,PIK3R2,RELA, |
| hsa05221 | Acute myeloid leukemia | 6 | 1.37E-03 | 1.85E-02 | 3.24E-01 | AKT1,RAF1,RPS6KB2,PIK3CD,PIK3R2,RELA |
| hsa04664 | Fc epsilon RI signaling pathway | 7 | 1.41E-03 | 1.85E-02 | 3.34E-01 | RAC2,AKT1,RAF1,LCP2,PIK3CD,PLCG1,PIK3R2 |
| hsa04722 | Neurotrophin signaling pathway | 9 | 1.67E-03 | 2.08E-02 | 3.95E-01 | AKT1,RAF1,ARHGDIA,PLCG1,RELA,MAP3K3,MAPKAPK2,PIK3CD,PIK3R2 |
| hsa04650 | Natural killer cell mediated cytotoxicity | 9 | 2.32E-03 | 2.73E-02 | 5.50E-01 | LCK,RAF1,HLA-C,PLCG1,RAC2,PTK2B,LCP2,PIK3CD,PIK3R2 |

**Supplementary Table 2D: Differentially reactive antigens, which hits Reactome associated molecules**

| **Pathway identifier** | **Pathway name** | **#Entities**  **found** | **#Entities**  **total** | **#Interactors**  **found** | **Submitted entities found** |
| --- | --- | --- | --- | --- | --- |
| R-HSA-450531 | Regulation of mRNA stability by proteins that bind AU-rich elements | 13 | 88 | 29 | Q14997;Q15008;O75888;P31749;P11940;Q9UNM6;Q06323;  P17980;P62987;P49137;P04792;P43686;P25787 |
| R-HSA-5663202 | Diseases of signal transduction | 26 | 384 | 47 | Q7KZF4;O00329;P06239;Q13438;O15164;P19174;  O60907;Q06323;Q9UBS0;P42224;P49815;P25787;  Q14997;O00459;Q15008;P04049;P30876;P31749;  Q9UNM6;Q14738;P17980;P51797;P62987;P43686;  P02675;P02751 |
| R-HSA-194138 | Signaling by VEGF | 26 | 347 | 32 | Q13813;P19174;P49023;Q06323;O43639;P49137;P25787;  Q14997;O00459;Q15008;P04049;P35968;P31749;Q9UNM6;  Q14738;P17980;Q01082;P18433;Q05513;P62987;Q14289;  P04792;P55160;P43686;P02675;P02751 |
| R-HSA-8866910 | TFAP2 (AP-2) family regulates transcription of growth factors and their receptors | 0 | 13 | 33 |  |
| R-HSA-8864260 | Transcriptional regulation by the AP-2 (TFAP2) family of transcription factors | 1 | 37 | 51 | P06748 |
| R-HSA-5687128 | MAPK6/MAPK4 signaling | 9 | 88 | 22 | Q14997;Q15008;P62987;Q9UNM6;Q06323;P17980;  P04792;P43686;P25787 |
| R-HSA-4420097 | VEGFA-VEGFR2 Pathway | 26 | 338 | 30 | Q13813;P19174;P49023;Q06323;O43639;P49137;  P25787;Q14997;O00459;Q15008;P04049;P35968;  P31749;Q9UNM6;Q14738;P17980;Q01082;P18433;  Q05513;P62987;Q14289;P04792;P55160;P43686;  P02675;P02751 |
| R-HSA-5683057 | MAPK family signaling cascades | 17 | 298 | 28 | Q14997;Q15008;P04049;P29597;Q13813;Q9UNM6;  Q06323;Q14738;Q01082;P17980;P18433;P62987;  P04792;P43686;P02675;P25787;P02751 |
| R-HSA-202403 | TCR signaling | 14 | 137 | 19 | Q14997;O00459;Q15008;P50552;P06239;P19174;  Q9UNM6;Q06323;P17980;Q04206;P62987;Q13094;  P43686;P25787 |
| R-HSA-450385 | Butyrate Response Factor 1 (BRF1) binds and destabilizes mRNA | 2 | 17 | 15 | P31749;P49137 |
| R-HSA-445144 | Signal transduction by L1 | 0 | 21 | 19 |  |
| R-HSA-1168372 | Downstream signaling events of B Cell Receptor (BCR) | 16 | 206 | 17 | Q14997;O00459;Q15008;O00329;P06239;P31749;  Q9UNM6;Q06323;Q14738;P17980;Q9UBS0;Q04206;  P62987;P49815;P43686;P25787 |
| R-HSA-1433557 | Signaling by SCF-KIT | 25 | 346 | 22 | Q14997;O00459;Q15008;P04049;O00329;P06239;  P07947;Q13813;Q92729;P31749;Q9UNM6;Q14738;  Q06323;P17980;Q01082;P18433;Q9UBS0;P42224;  P62987;P49815;P43686;P02675;P25787;P02751 |
| R-HSA-5358346 | Hedgehog ligand biogenesis | 10 | 65 | 1 | Q14997;Q15008;Q13438;P62987;Q9UNM6;Q06323;  P17980;P43686;P07237;P25787 |
| R-HSA-450408 | AUF1 (hnRNP D0) binds and destabilizes mRNA | 10 | 56 | 8 | Q14997;Q15008;P62987;P11940;Q9UNM6;Q06323;  P17980;P04792;P43686;P25787 |
| R-HSA-1169091 | Activation of NF-kappaB in B cells | 9 | 67 | 3 | Q14997;Q15008;Q04206;P62987;Q9UNM6;Q06323;  P17980;P43686;P25787 |
| R-HSA-2467813 | Separation of Sister Chromatids | 19 | 184 | 1 | Q14997;Q15008;Q7Z5K2;P49454;Q96BT3;P68363;  Q9UNM6;P57740;Q06323;Q14738;P17980;Q7L7X3;  P55735;Q9H410;P62987;Q9GZM8;O14777;P43686;P25787 |
| R-HSA-68949 | Orc1 removal from chromatin | 11 | 71 | 0 | Q14997;Q15008;Q9UM11;P62987;P33993;Q9UNM6;  Q06323;P17980;P43686;P25787;Q9H211 |
| R-HSA-193639 | p75NTR signals via NF-kB | 2 | 17 | 12 | Q04206;P62987 |
| R-HSA-69017 | CDK-mediated phosphorylation and removal of Cdc6 | 8 | 51 | 1 | Q14997;Q15008;P62987;Q9UNM6;Q06323;P17980;P43686;P25787 |
