# Supplementary material for: IgG based immunome analyses of breast cancer patients reveal underlying signaling pathways
Source: Oncotarget. 2019 May 28;10(37):3491–505. doi: 10.18632/oncotarget.26834 (PMC6544406; doi:10.18632/oncotarget.26834)
Supplement: Supplementary file 4 [file oncotarget-10-3491-s004.docx]

**Supplementary Table 3: Genes of the enriched genomic regions, according to GSEA analysis**

| Overlap Results |  |  |  |  |  |  |
| --- | --- | --- | --- | --- | --- | --- |
|  |  |  |  |  |  |  |
| Collection(s): | C1 |  |  |  |  |  |
| # overlaps shown: | 4 |  |  |  |  |  |
| # genesets in collections: | 326 |  |  |  |  |  |
| # genes in comparison (n): | 507 |  |  |  |  |  |
| # genes in universe (N): | 45956 |  |  |  |  |  |
|  |  |  |  |  |  |  |
| Gene Set Name | # Genes in Gene Set (K) | Description | # Genes in Overlap (k) | k/K | p-value | FDR q-value |
| chr3p21 | 271 | Genes in cytogenetic band chr3p21 | 14 | 0.0517 | 2.36E-06 | 7.70E-04 |
| chr19q13 | 948 | Genes in cytogenetic band chr19q13 | 27 | 0.0285 | 9.21E-06 | 1.50E-03 |
| chr19p13 | 645 | Genes in cytogenetic band chr19p13 | 20 | 0.031 | 4.15E-05 | 4.51E-03 |
| chr1p36 | 504 | Genes in cytogenetic band chr1p36 | 15 | 0.0298 | 5.63E-04 | 4.59E-02 |
|  |  |  |  |  |  |  |
|  |  |  |  |  |  |  |
| Gene/Gene Set Overlap Matrix |  |  |  |  |  |  |
|  |  |  |  |  |  |  |
| Entrez Gene Id | Gene Symbol | Gene Description | chr3p21 | chr19q13 | chr19p13 | chr1p36 |
| 11188 | NISCH | nischarin | chr3p21 |  |  |  |
| 11344 | TWF2 | twinfilin, actin-binding protein, homolog 2 (Drosophila) | chr3p21 |  |  |  |
| 1154 | CISH | cytokine inducible SH2-containing protein | chr3p21 |  |  |  |
| 22937 | SCAP | SREBF chaperone | chr3p21 |  |  |  |
| 23166 | STAB1 | stabilin 1 | chr3p21 |  |  |  |
| 25930 | PTPN23 | protein tyrosine phosphatase, non-receptor type 23 | chr3p21 |  |  |  |
| 275 | AMT | aminomethyltransferase | chr3p21 |  |  |  |
| 2771 | GNAI2 | guanine nucleotide binding protein (G protein), alpha inhibiting activity polypeptide 2 | chr3p21 |  |  |  |
| 29072 | SETD2 | SET domain containing 2 | chr3p21 |  |  |  |
| 29925 | GMPPB | GDP-mannose pyrophosphorylase B | chr3p21 |  |  |  |
| 378 | ARF4 | ADP-ribosylation factor 4 | chr3p21 |  |  |  |
| 51460 | SFMBT1 | Scm-like with four mbt domains 1 | chr3p21 |  |  |  |
| 55193 | PBRM1 | polybromo 1 | chr3p21 |  |  |  |
| 5859 | QARS | glutaminyl-tRNA synthetase | chr3p21 |  |  |  |
| 11100 | HNRNPUL1 | heterogeneous nuclear ribonucleoprotein U-like 1 |  | chr19q13 |  |  |
| 114823 | LENG8 | leukocyte receptor cluster (LRC) member 8 |  | chr19q13 |  |  |
| 1155 | TBCB | tubulin folding cofactor B |  | chr19q13 |  |  |
| 126133 | ALDH16A1 | aldehyde dehydrogenase 16 family, member A1 |  | chr19q13 |  |  |
| 160 | AP2A1 | adaptor-related protein complex 2, alpha 1 subunit |  | chr19q13 |  |  |
| 25865 | PRKD2 | protein kinase D2 |  | chr19q13 |  |  |
| 26121 | PRPF31 | PRP31 pre-mRNA processing factor 31 homolog (S. cerevisiae) |  | chr19q13 |  |  |
| 29888 | STRN4 | striatin, calmodulin binding protein 4 |  | chr19q13 |  |  |
| 29903 | CCDC106 | coiled-coil domain containing 106 |  | chr19q13 |  |  |
| 3770 | KCNJ14 | potassium inwardly-rectifying channel, subfamily J, member 14 |  | chr19q13 |  |  |
| 402665 | IGLON5 | IgLON family member 5 |  | chr19q13 |  |  |
| 5050 | PAFAH1B3 | platelet-activating factor acetylhydrolase 1b, catalytic subunit 3 (29kDa) |  | chr19q13 |  |  |
| 5296 | PIK3R2 | phosphoinositide-3-kinase, regulatory subunit 2 (beta) |  | chr19q13 |  |  |
| 55101 | ATP5SL | ATP5S-like |  | chr19q13 |  |  |
| 5704 | PSMC4 | proteasome (prosome, macropain) 26S subunit, ATPase, 4 |  | chr19q13 |  |  |
| 57348 | TTYH1 | tweety homolog 1 (Drosophila) |  | chr19q13 |  |  |
| 57677 | ZFP14 | zinc finger protein 14 homolog (mouse) |  | chr19q13 |  |  |
| 602 | BCL3 | B-cell CLL/lymphoma 3 |  | chr19q13 |  |  |
| 6829 | SUPT5H | suppressor of Ty 5 homolog (S. cerevisiae) |  | chr19q13 |  |  |
| 7408 | VASP | vasodilator-stimulated phosphoprotein |  | chr19q13 |  |  |
| 7593 | MZF1 | myeloid zinc finger 1 |  | chr19q13 |  |  |
| 79784 | MYH14 | myosin, heavy chain 14, non-muscle |  | chr19q13 |  |  |
| 81 | ACTN4 | actinin, alpha 4 |  | chr19q13 |  |  |
| 84446 | BRSK1 | BR serine/threonine kinase 1 |  | chr19q13 |  |  |
| 90338 | ZNF160 | zinc finger protein 160 |  | chr19q13 |  |  |
| 90485 | ZNF835 | zinc finger protein 835 |  | chr19q13 |  |  |
| 92799 | SHKBP1 | SH3KBP1 binding protein 1 |  | chr19q13 |  |  |
| 10212 | DDX39A | DEAD (Asp-Glu-Ala-Asp) box polypeptide 39A |  |  | chr19p13 |  |
| 23149 | FCHO1 | FCH domain only 1 |  |  | chr19p13 |  |
| 29911 | HOOK2 | hook homolog 2 (Drosophila) |  |  | chr19p13 |  |
| 4650 | MYO9B | myosin IXB |  |  | chr19p13 |  |
| 4670 | HNRNPM | heterogeneous nuclear ribonucleoprotein M |  |  | chr19p13 |  |
| 51343 | FZR1 | fizzy/cell division cycle 20 related 1 (Drosophila) |  |  | chr19p13 |  |
| 54862 | CC2D1A | coiled-coil and C2 domain containing 1A |  |  | chr19p13 |  |
| 55702 | CCDC94 | coiled-coil domain containing 94 |  |  | chr19p13 |  |
| 55723 | ASF1B | ASF1 anti-silencing function 1 homolog B (S. cerevisiae) |  |  | chr19p13 |  |
| 57130 | ATP13A1 | ATPase type 13A1 |  |  | chr19p13 |  |
| 57693 | ZNF317 | zinc finger protein 317 |  |  | chr19p13 |  |
| 5802 | PTPRS | protein tyrosine phosphatase, receptor type, S |  |  | chr19p13 |  |
| 5976 | UPF1 | UPF1 regulator of nonsense transcripts homolog (yeast) |  |  | chr19p13 |  |
| 7297 | TYK2 | tyrosine kinase 2 |  |  | chr19p13 |  |
| 7311 | UBA52 | ubiquitin A-52 residue ribosomal protein fusion product 1 |  |  | chr19p13 |  |
| 79958 | DENND1C | DENN/MADD domain containing 1C |  |  | chr19p13 |  |
| 81794 | ADAMTS10 | ADAM metallopeptidase with thrombospondin type 1 motif, 10 |  |  | chr19p13 |  |
| 8666 | EIF3G | eukaryotic translation initiation factor 3, subunit G |  |  | chr19p13 |  |
| 93145 | OLFM2 | olfactomedin 2 |  |  | chr19p13 |  |
| 9592 | IER2 | immediate early response 2 |  |  | chr19p13 |  |
| 11079 | RER1 | RER1 retention in endoplasmic reticulum 1 homolog (S. cerevisiae) |  |  |  | chr1p36 |
| 1185 | CLCN6 | chloride channel 6 |  |  |  | chr1p36 |
| 1676 | DFFA | DNA fragmentation factor, 45kDa, alpha polypeptide |  |  |  | chr1p36 |
| 23352 | UBR4 | ubiquitin protein ligase E3 component n-recognin 4 |  |  |  | chr1p36 |
| 2582 | GALE | UDP-galactose-4-epimerase |  |  |  | chr1p36 |
| 26155 | NOC2L | nucleolar complex associated 2 homolog (S. cerevisiae) |  |  |  | chr1p36 |
| 26270 | FBXO6 | F-box protein 6 |  |  |  | chr1p36 |
| 27237 | ARHGEF16 | Rho guanine nucleotide exchange factor (GEF) 16 |  |  |  | chr1p36 |
| 3339 | HSPG2 | heparan sulfate proteoglycan 2 |  |  |  | chr1p36 |
| 5293 | PIK3CD | phosphoinositide-3-kinase, catalytic, delta polypeptide |  |  |  | chr1p36 |
| 54998 | AURKAIP1 | aurora kinase A interacting protein 1 |  |  |  | chr1p36 |
| 5590 | PRKCZ | protein kinase C, zeta |  |  |  | chr1p36 |
| 57470 | LRRC47 | leucine rich repeat containing 47 |  |  |  | chr1p36 |
| 6248 | RSC1A1 | regulatory solute carrier protein, family 1, member 1 |  |  |  | chr1p36 |
| 864 | RUNX3 | runt-related transcription factor 3 |  |  |  | chr1p36 |
