# Supplementary material for: IgG based immunome analyses of breast cancer patients reveal underlying signaling pathways
Source: Oncotarget. 2019 May 28;10(37):3491–505. doi: 10.18632/oncotarget.26834 (PMC6544406; doi:10.18632/oncotarget.26834)
Supplement: Supplementary file 5 [file oncotarget-10-3491-s005.docx]

**Supplementary Table 4: Clinical features of breast cancer patients**

| Sample | Batch | STUDY_CpG360SERUM | Age of Diagonosis | Menopausenstatus | Histology | Tumor Staging | Lymph node | Grading | Metastasis status | Estrogen Receptor | Progestron Receptor | Hercep Test |
| --- | --- | --- | --- | --- | --- | --- | --- | --- | --- | --- | --- | --- |
| P11 | 1 | 7_M0_G2 | 55 | Post - Menopause | Invasive ductal carcinoma | pT1a | pN0 | G2 | 0 | Negative | Negative | Positive |
| P25 | 1 | 8_M0_G3 | 57 | Post - Menopause | Invasive ductal carcinoma | pT1b | pN0 | G3 | MO | Strong | Negative | Positive |
| P26 | 1 | 6_M0_G1 | 52 | Post - Menopause | Invasive ductal carcinoma | pT2 | pN1a | G1 | MO | Moderate | Negative | Positive |
| P59 | 1 | 7_M0_G2 | 70 | Post - Menopause | Invasive ductal carcinoma | pT1c | pN0 | G2 | MO | Strong | Negative | Positive |
| P65 | 1 | 7_M0_G2 | 64 | Post - Menopause | Invasive ductal carcinoma | pT1c | pN0 | G2 | MO | Negative | Negative | Positive |
| P128 | 1 | 8_M0_G3 | 64 | Post - Menopause | Invasive ductal carcinoma | pT1a | PN1 | G3 | MO | Moderate | Negative | Positive |
| P137 | 1 | 8_M0_G3 | 53 | Post - Menopause | Invasive ductal carcinoma | pT1c | pN2a | G3 | 0 | Negative | Negative | Positive |
| P138 | 1 | 8_M0_G3 | 59 | Post - Menopause | Invasive ductal carcinoma | pT1c | pN2 | G3 | 0 | Strong | Negative | Positive |
| P161 | 1 | 8_M0_G3 | 32 | Pre - Menopause | Invasive ductal carcinoma | pT1c | pN2a | G3 | 0 | Moderate | Negative | Positive |
| P170 | 1 | 7_M0_G2 | 63 | Post - Menopause | Invasive ductal carcinoma | pT2 | pN2a | G2 | 0 | Moderate | Negative | Positive |
| P191 | 1 | 8_M0_G3 | 51 | Pre - Menopause | Invasive ductal carcinoma | pT1mic | pN3 | G3 | 0 | Negative | Negative | Positive |
| P258 | 1 | 4_M1 | 40 | n/a | Invasive ductal carcinoma | pT1c | pN3 | G3 | MO | Negative | Negative | Positive |
| P5 | 2 | 3_relapse | 62 | Post - Menopause | Invasive lobular carcinoma (10-20%) | pT1c | pN0 | G2 | MO | Strong | Negative | Negative |
| P202 | 2 | 8_M0_G3 | 78 | Post - Menopause | Invasive ductal carcinoma | pT2 | pN0 | G3 | 0 | Weak | Moderate | Positive |
| P224 | 2 | 5_sporM0_CIS | 56 | Pre - Menopause | Carcinoma, intraductal, noninvasive | pTis | pNX | G1 | MX | Moderate | Moderate | Negative |
| P248 | 2 | 8_M0_G3 | 44 | Pre - Menopause | Invasive ductal carcinoma | pT2 | pN1a | G3 | 0 | Moderate | Negative | Negative |
| P257 | 2 | 7_M0_G2 | 79 | Post - Menopause | Invasive ductal carcinoma | pT1c | pN0 | G2 | 0 | Strong | Strong | Negative |
| P268 | 2 | 7_M0_G2 | 83 | Post - Menopause | Invasive ductal carcinoma | pT1c | pN0 | G2 | 0 | Strong | Weak | Negative |
| P279 | 2 | 8_M0_G3 | 56 | Post - Menopause | n/a | pT1c | pN0 | G3 | 0 | Negative | Negative | Positive |
| P280 | 2 | 6_M0_G1 | 43 | Pre - Menopause | Carcinoma, intraductal, noninvasive | n/a | n/a | G1 | n/a | n/a | n/a | n/a |
| P281 | 2 | 8_M0_G3 | 60 | Post - Menopause | Invasive ductal carcinoma | pT1b | pN0 | G3 | 0 | Weak | Negative | Positive |
| P307 | 2 | 7_M0_G2 | 41 | Pre - Menopause | Invasive ductal carcinoma | pT1c | pN0 | G2 | 0 | Moderate | Negative | Negative |
| P325 | 2 | 4_M1 | 63 | Post - Menopause | n/a | pT2 | PN1 | G3 | M1 | Moderate | Negative | Negative |
| P329 | 2 | 3_relapse | 67 | Post - Menopause | Invasive lobular carcinoma (10-20%) | pT1c | n/a | G2 | 0 | Strong | Strong | Negative |
| P8 | 3 | 6_M0_G1 | 63 | Post - Menopause | Invasive ductal carcinoma | pT1c | pN1mi | G1 | MO | Strong | Weak | Negative |
| P48 | 3 | 4_M1 | 0 | Post - Menopause | Invasive ductal carcinoma | pT1c | pNX | G3 | M1 | Positive | Negative | Negative |
| P60 | 3 | 3_relapse | 53 | Post - Menopause | Invasive ductal carcinoma | pT1b | pN1bi | G2 | 0 | Negative | Moderate | Negative |
| P97 | 3 | no data | n/a | n/a | n/a | n/a | n/a | n/a | n/a | n/a | n/a | n/a |
| P106 | 3 | 6_M0_G1 | 78 | Post - Menopause | Adenoid cystic carcinoma | pT1mic | pN0 | G1 | MO | Strong | Strong | Negative |
| P132 | 3 | 6_M0_G1 | 70 | Post - Menopause | Invasive lobular carcinoma (10-20%) | pT1c | pN0 | G1 | MO | Strong | Weak | Negative |
| P136 | 3 | 6_M0_G1 | 78 | Post - Menopause | n/a | pT1c | pN0 | G1 | 0 | Moderate | Weak | Negative |
| P164 | 3 | 7_M0_G2 | 45 | n/a | Invasive lobular carcinoma (10-20%) | pT4b | pN3 | G2 | 0 | Moderate | Strong | Negative |
| P188 | 3 | 3_relapse | 55 | Post - Menopause | Invasive ductal carcinoma | pT2 | pN0 | G3 | MX | Strong | Negative | Positive |
| P192 | 3 | 3_relapse | 53 | Post - Menopause | Invasive ductal carcinoma | pT1c | pN0 | G3 | 0 | Negative | Negative | Negative |
| P207 | 3 | 5_sporM0_CIS | 70 | Post - Menopause | Carcinoma, intraductal, noninvasive | pTis | pN0 | G3 | 0 | Negative | Negative | Positive |
| P208 | 3 | 7_M0_G2 | 80 | Post - Menopause | Invasive lobular carcinoma (10-20%) | pT2 | pN0 | G2 | 0 | Moderate | Negative | Positive |
| P222 | 4 | 6_M0_G1 | 56 | Post - Menopause | Invasive ductal carcinoma | pT1 | pN0 | G1 | 0 | Moderate | Positive | Negative |
| P225 | 4 | 5_sporM0_CIS | 46 | Pre - Menopause | Carcinoma, intraductal, noninvasive | pTis | pNX | G1 | MX | Moderate | Moderate | Negative |
| P242 | 4 | 5_sporM0_CIS | 70 | Post - Menopause | Carcinoma, intraductal, noninvasive | pTis | pN0 | G2 | 0 | Strong | Strong | Negative |
| P243 | 4 | 7_M0_G2 | 40 | Pre - Menopause | Invasive ductal carcinoma | pT1c | pN0 | G2 | 0 | Moderate | Negative | Positive |
| P254 | 4 | 4_M1 | 49 | Pre - Menopause | Invasive ductal carcinoma | pT1b | pN1a | G3 | MX | Negative | Negative | Negative |
| P262 | 4 | 8_M0_G3 | 40 | Pre - Menopause | Invasive ductal carcinoma | pT1c | pN1b | G3 | 0 | Moderate | Moderate | Negative |
| P266 | 4 | 7_M0_G2 | 42 | Pre - Menopause | Invasive ductal carcinoma | pTis | pN1a | G2 | 0 | Moderate | Moderate | Positive |
| P267 | 4 | 5_sporM0_CIS | 77 | Post - Menopause | Carcinoma, intraductal, noninvasive | pT1mic | pNX | G1 | 0 | Strong | Strong | Negative |
| P269 | 4 | 8_M0_G3 | 70 | Post - Menopause | Invasive ductal carcinoma | pT2 | pN0 | G3 | 0 | Moderate | Negative | Negative |
| P273 | 4 | 4_M1 | 27 | Pre - Menopause | Invasive ductal carcinoma | pT2 | pN3 | G3 | 0 | Negative | Negative | Negative |
| P275 | 4 | 4_M1 | 55 | Post - Menopause | Invasive ductal carcinoma | pT2 | pN2a | G2 | M1 | Strong | Negative | Negative |
| P276 | 4 | 8_M0_G3 | 46 | Pre - Menopause | Invasive ductal carcinoma | pT1a | pN0 | G3 | 0 | Negative | Negative | Positive |
| P114 | 5 | 4_M1 | 72 | Post - Menopause | Invasive lobular carcinoma (10-20%) | pT2 | PN1 | G2 | M1 | Strong | Moderate | Positive |
| P117 | 5 | 7_M0_G2 | 53 | Pre - Menopause | Invasive ductal carcinoma | pT1c | PN1 | G2 | MO | Strong | Negative | Negative |
| P129 | 5 | 6_M0_G1 | 56 | Post - Menopause | Invasive lobular carcinoma (10-20%) | pT1b | pN0 | G1 | MO | Positive | Moderate | n/a |
| P130 | 5 | 8_M0_G3 | 74 | Post - Menopause | Invasive ductal carcinoma | n/a | pN0 | G3 | MO | Moderate | Negative | Negative |
| P135 | 5 | 6_M0_G1 | 52 | Post - Menopause | Invasive ductal carcinoma | pT2 | pN0 | G1 | MO | Strong | Negative | Negative |
| P141 | 5 | no data | n/a | n/a | n/a | n/a | n/a | n/a | n/a | n/a | n/a | n/a |
| P142 | 5 | 6_M0_G1 | 45 | Pre - Menopause | Invasive ductal carcinoma | pT1c | PN1 | G1 | 0 | Strong | Strong | Negative |
| P15 | 5 | 3_relapse | 44 | Post - Menopause | Invasive ductal carcinoma | pT1 | pN0 | G1 | MO | Positive | Negative | Negative |
| P168 | 5 | 5_sporM0_CIS | 62 | Post - Menopause | Carcinoma, intraductal, noninvasive | pTis | pN0 | G2 | MO | Strong | Strong | Negative |
| P173 | 5 | 6_M0_G1 | 75 | Post - Menopause | Invasive ductal carcinoma | pT2 | pN0 | G1 | MO | Strong | Strong | Negative |
| P176 | 5 | 6_M0_G1 | 64 | Post - Menopause | Adenoid cystic carcinoma | pT1c | pN0 | G1 | MO | Negative | Negative | Negative |
| P195 | 5 | 5_sporM0_CIS | 49 | Post - Menopause | Carcinoma, intraductal, noninvasive | pTis | pN0 | G1 | 0 | Strong | Negative | Negative |
| P196 | 6 | 5_sporM0_CIS | 60 | Post - Menopause | Carcinoma, intraductal, noninvasive | pTis | n/a | G2 | 0 | Strong | Moderate | Negative |
| P201 | 6 | 5_sporM0_CIS | 44 | Pre - Menopause | Carcinoma, intraductal, noninvasive | pTis | pNX | G1 | MX | Moderate | Moderate | Negative |
| P204 | 6 | 6_M0_G1 | 74 | Post - Menopause | Invasive ductal carcinoma | pT1a | pN0 | G1 | 0 | Negative | Negative | Negative |
| P21 | 6 | 4_M1 | 57 | n/a | Muzinous carcinoma | pTX | pNX | n/a | M1 | n/a | n/a | n/a |
| P211 | 6 | 5_sporM0_CIS | 42 | Pre - Menopause | Carcinoma, intraductal, noninvasive | pT1c | pN0 | G1 | 0 | Strong | Strong | Negative |
| P212 | 6 | 8_M0_G3 | 47 | n/a | Invasive ductal carcinoma | pT2 | pN0 | G3 | 0 | Negative | Negative | Negative |
| P217 | 6 | 4_M1 | 46 | Pre - Menopause | n/a | pT2 | pN2a | G3 | 0 | Strong | Moderate | Negative |
| P290 | 6 | 5_sporM0_CIS | 53 | n/a | Carcinoma, intraductal, noninvasive | pTis | pN0 | G2 | MO | Negative | Negative | Negative |
| P24 | 6 | 3_relapse | 53 | Post - Menopause | Invasive ductal carcinoma | pT1c | pNX | G2 | 0 | Negative | Negative | Negative |
| P244 | 6 | 8_M0_G3 | 51 | Post - Menopause | Invasive ductal carcinoma | pT1b | n/a | G3 | 0 | Negative | Negative | Positive |
| P247 | 6 | 8_M0_G3 | 50 | Peri - Menopause | Invasive ductal carcinoma | pT1a | pN1a | G3 | 0 | Negative | Negative | Negative |
| P251 | 6 | 8_M0_G3 | 60 | Post - Menopause | Invasive ductal carcinoma | pT1c | pN0 | G3 | 0 | Negative | Negative | Negative |
| P265 | 6 | 8_M0_G3 | 35 | Pre - Menopause | Invasive ductal carcinoma | pT2 | pN0 | G3 | 0 | Negative | Negative | Negative |
| P270 | 6 | 4_M1 | 58 | Post - Menopause | Invasive ductal carcinoma | pT1c | pN1a | G3 | M1 | Moderate | Negative | Negative |
| P278 | 6 | 4_M1 | 48 | Pre - Menopause | n/a | pT2 | pN1a | G3 | M1 | Weak | Weak | Negative |
| P282 | 6 | 5_sporM0_CIS | 53 | n/a | Carcinoma, intraductal, noninvasive | pTis | pNX | G3 | MX | Strong | Negative | Positive |
| P283 | 6 | 7_M0_G2 | 43 | Pre - Menopause | Invasive ductal carcinoma | pT1c | n/a | G2 | 0 | Moderate | Moderate | Positive |
| P286 | 6 | 5_sporM0_CIS | 58 | n/a | Carcinoma, intraductal, noninvasive | pTis | pN0 | G3 | 0 | Negative | Negative | Positive |
